# Functional enrichment of alternative splicing events with NEASE reveals insights into tissue identity and diseases

**DOI:** 10.1186/s13059-021-02538-1

**Published:** 2021-12-02

**Authors:** Zakaria Louadi, Maria L. Elkjaer, Melissa Klug, Chit Tong Lio, Amit Fenn, Zsolt Illes, Dario Bongiovanni, Jan Baumbach, Tim Kacprowski, Markus List, Olga Tsoy

**Affiliations:** 1 Chair of Experimental Bioinformatics, TUM School of Life Sciences, Technical University of Munich, 85354 Freising, Germany; 2 Institute for Computational Systems Biology, University of Hamburg, Notkestrasse 9, 22607 Hamburg, Germany; 3 Department of Neurology, Odense University Hospital, Odense, Denmark; 4 Institute of Clinical Research, University of Southern Denmark, Odense, Denmark; 5 Institute of Molecular Medicine, University of Southern Denmark, Odense, Denmark; 6 Department of Internal Medicine I, School of Medicine, University hospital rechts der Isar, Technical University of Munich, Munich, Germany; 7 German Center for Cardiovascular Research (DZHK), Partner Site Munich Heart Alliance, Munich, Germany; 8 Department of Cardiovascular Medicine, Humanitas Clinical and Research Center IRCCS and Humanitas University, Rozzano, Milan, Italy; 9 Institute of Mathematics and Computer Science, University of Southern Denmark, Campusvej 55, 5000 Odense, Denmark; 10 Division Data Science in Biomedicine, Peter L. Reichertz Institute for Medical Informatics of Technische Universität Braunschweig and Hannover Medical School, Braunschweig, Germany; 11 Braunschweig Integrated Centre of Systems Biology (BRICS), TU Braunschweig, Braunschweig, Germany

**Keywords:** Alternative splicing, Differential splicing, Functional enrichment, Systems biology, Protein-protein interactions, Disease pathways, Platelet activation, Multiple sclerosis, Dilated cardiomyopathy

## Abstract

Alternative splicing (AS) is an important aspect of gene regulation. Nevertheless, its role in molecular processes and pathobiology is far from understood. A roadblock is that tools for the functional analysis of AS-set events are lacking. To mitigate this, we developed NEASE, a tool integrating pathways with structural annotations of protein-protein interactions to functionally characterize AS events. We show in four application cases how NEASE can identify pathways contributing to tissue identity and cell type development, and how it highlights splicing-related biomarkers. With a unique view on AS, NEASE generates unique and meaningful biological insights complementary to classical pathways analysis.

**Supplementary Information:**

The online version contains supplementary material available at 10.1186/s13059-021-02538-1.

## Background

Alternative splicing (AS) boosts transcript diversity in human cells [1] and thus contributes to tissue identity [2], cell development [3], and pathology in, e.g., cardiomyopathy [4], muscular dystrophy [5], or autoimmune diseases [6]. It is estimated that up to 30% of disease-associated genetic variants affect splicing [7]. RNA sequencing technologies (RNA-seq) allow the quantification of different types of AS events and detect splicing abnormalities in disorders. However, RNA-seq utility is currently limited by our incomplete understanding of the functional role of specific exons or the transcripts they contribute to.

A major challenge in AS analysis is the functional interpretation of a set of events, including isoform switching events and differentially spliced exons. The usual approach is to perform gene set enrichment or overrepresentation analysis [8–10]. This approach treats all genes affected by AS equally, neglecting that some AS events may not be functionally relevant at the protein level [11] or result from noise in the splicing machinery [12]. Furthermore, functional differences between protein isoforms remain uncertain in many cases. A promising strategy to identify relevant AS events is to focus on those that lead to meaningful changes in the protein structure. Recent studies have shown that AS has the potential to rewire protein-protein interactions by affecting the inclusion of domain families [13] and linear motifs [14] or by activating nonsense-mediated decay [15].

This motivated the creation of databases and tools that predict the consequences of individual AS events or isoform switches. IsoformSwitchAnalyzeR [16], tappAS [17], DoChaP [18], and Spada [19] support transcript-level (as opposed to exon-level) analysis to identify isoform switches and their impact on the translation and the resulting isoforms features, such as domains, motifs, and non-coding sites. Exon Ontology [20] and DIGGER [21] support exon-level analysis to identify exon skipping events and their possible impact on the protein structure and function. Spada and DIGGER further consider the impact of AS on protein-protein interactions.

Most existing tools allow investigating AS-driven changes in an explorative fashion but tools for systematic analysis of functional effects of AS are lacking. Exon Ontology performs statistical tests to identify enriched features within a set of skipped exons. One example is domain families affected by AS across proteins more frequently than expected. However, none of the existing tools offer a systems biology view to specifically highlight functional consequences of AS events.

To tackle these limitations, we developed the first tool for functional enrichment of AS events. NEASE (Network-based Enrichment method for AS Events) first detects protein domains affected by AS and then uses protein-protein interactions (PPI) integrated with domain-domain interactions (DDI) [21], residue-level, and domain-motif interactions (DMI) [22] to identify interaction partners likely affected by AS. Next, it employs an edge-level hypergeometric test for gene set overrepresentation analysis. This approach is new in the way genes are selected for the enrichment test. Rather than considering only differentially spliced or expressed genes, which is currently the most common strategy, NEASE uses network information to select genes that are likely affected in the interactome. This is also superior to a simple network enrichment analysis, as we consider only those edges for which an AS contribution seems relevant and for which false positive results are less likely. We evaluated NEASE using multiple datasets from both healthy and disease cohorts. We show that the NEASE approach complements gene-level enrichment, and even outperforms it in scenarios where gene-level enrichment fails to find relevant pathways. Moreover, NEASE generates unique and meaningful biological insights on the exact impact of AS. Furthermore, since the statistical approach is network-based, NEASE can prioritize (differentially) spliced genes and find new disease biomarkers candidates in case of aberrant splicing. The NEASE Python package, freely available at https://github.com/louadi/NEASE, provides multiple functions for a deeper analysis and visualization of affected protein domains, edges, and pathways (individually or as a set).

## Results

### Overview of NEASE

**Fig. 1:**
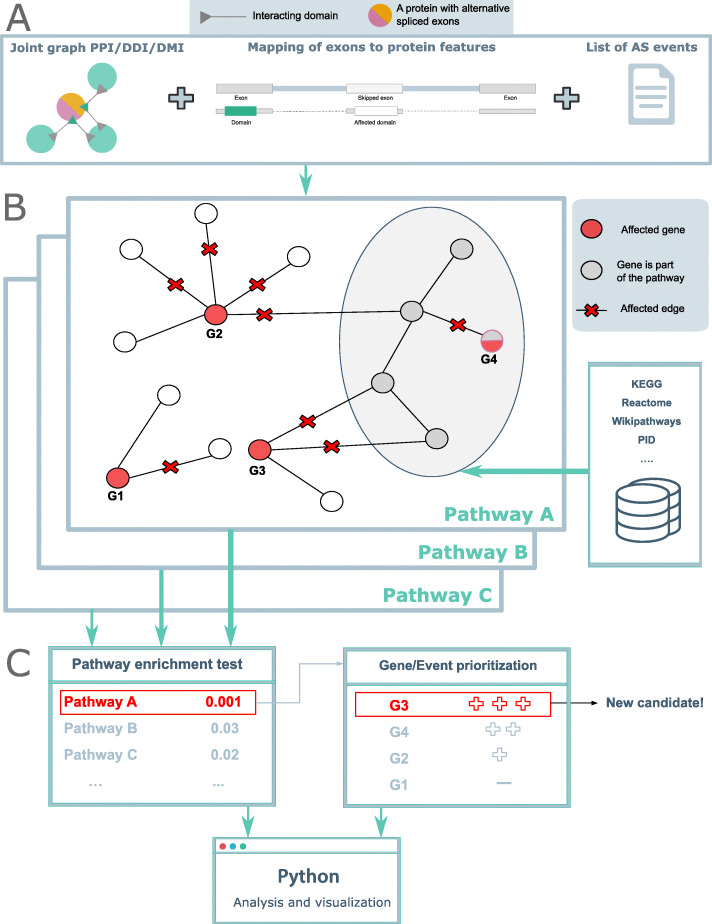
Overview of NEASE. **A** Annotated exons are mapped to Pfam domains, motifs, and residues. The joint graph of PPIs, DDIs, DMIs, and co-resolved structure is used to identify the interactions mediated by these features. **B** For a list of exons/events, NEASE identifies interactions mediated by the spliced protein features and pathways that are significantly affected by those interactions. **C** NEASE provides a corrected *p* value, in addition to an enrichment score (NEASE Score) for every pathway (see the “Methods” section). The user can further focus on an individual pathway, where NEASE can prioritize genes and find new biomarkers. In this example, the gene G3 was not part of the enriched pathway A but it has the largest number of affected interactions with genes from the pathway

NEASE uses a hybrid approach that combines biological pathways with PPIs and DDIs to perform functional enrichment of AS. First, we use the structural annotation of known isoforms by mapping protein domains from the Pfam database [23] to the corresponding exons (Fig. 1A). Second, we construct a structural joint graph as previously reported [21] by enriching the BioGRID PPI [24] with DDIs (from DOMINE [25] and 3did [26]), DMIs from the Eukaryotic Linear Motif resource (ELM) [22], and interface residues from the Protein Data Bank (PDB) [27] (see Methods). In the joint graph, protein features such as domains, motifs, and residues are mapped to their mediated interactions. Thus, NEASE provides an exon-centric view of the interactome and addresses the limited exon-level annotation. Exons are represented by the features they encode, and interactions between features are represented by edges. In this way, the impact of AS can be seen as an edgetic change in the network. Analyzed AS events are viewed as a set of affected edges that represent gained or lost PPIs.

We then perform statistical tests to find enriched pathways and most likely responsible genes (Fig. 1B). Following, (differential) splicing analysis, a one-sided hypergeometric test is used to test for enrichment of a given pathway or gene set by considering all edges affected by AS in an experiment. A similar test is applied for each spliced gene to prioritize the most relevant events/genes that are affecting a pathway. We further introduce a weighted score (NEASE score) that penalizes hub nodes that are more likely to be connected to the pathway of interest by chance. Notably, this approach also considers genes that are not part of the existing pathway definition but show a significant number of interactions with the pathway, highlighting new putative biomarkers (see Methods and Additional file 1: Figure S6, for details).

The Python package provides an interactive analysis. Using a list of exons or events, users can run a general enrichment on 12 different pathway databases (collected from the ConsensusPathDB resource [28]), followed up by a specific analysis and visualization for a single affected pathway or module of interest (Fig. 1C). To provide analysis for individual isoforms and events, we linked NEASE to our previously developed database DIGGER, which provides an isoform- and exon-centric view of the interactome [21].

To check if the structurally annotated PPI is more biased to hubs than the standard PPI network, we computed the node degree distribution of the network before and after filtering for the structure evidence. As shown in Additional file 1: Figure S1, the two histograms show similar trends with an overall smaller number of edges in the structurally annotated PPI. The latter has a maximum node degree equal to 424, compared to 2887 in the full PPI. This observation shows that the structurally annotated PPI does not increase the bias towards hub genes of the interactome.

### NEASE gives insights into the role of the muscle- and neural-specific exons

**Fig. 2:**
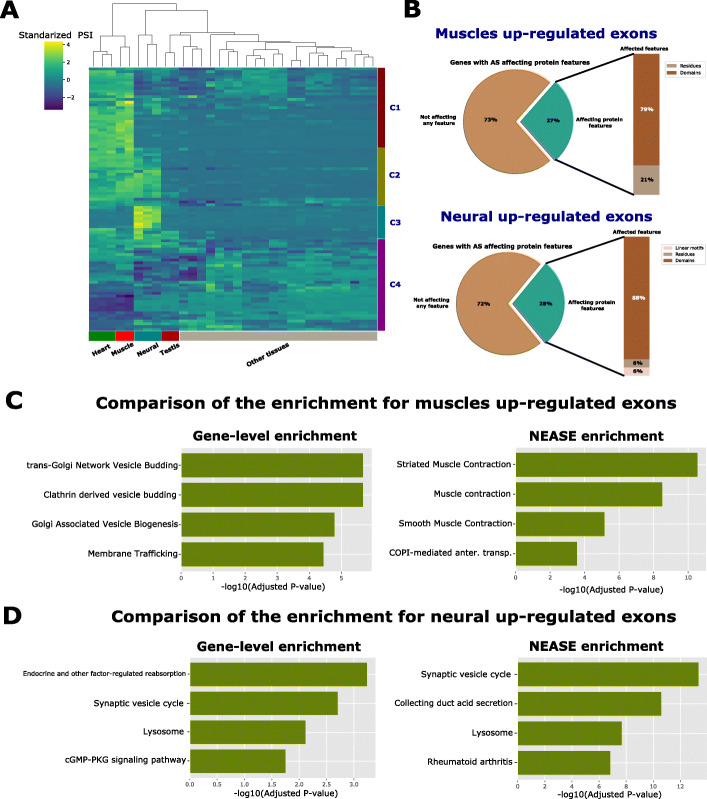
Analysis of tissue-specific exons. **A** Heatmap and hierarchical clustering of standardized PSI values obtained from VastDB. The heatmap only shows events with a standard deviation of PSI values ≥ 20. The heatmap shows that clusters of exons upregulated in neural tissues and muscle/heart tissues are dominant (clusters C1 and C3). **B** NEASE analysis shows that 28% and 27% for both neural and muscle upregulated exons, respectively, are encoding protein features: domains, linear motifs, and residues. For these subgroups of events, the exact protein complexes involved can be identified, and NEASE enrichment can be performed. **C**, **D** Comparison between gene-level enrichment and NEASE enrichment for the two sets of exons

Recent studies suggest that the regulation of AS occurs in a tissue-specific manner and leads to remodeling of protein-protein interactions [29]. Understanding the functional impact of co-regulated exons is critical in understanding gene regulation. We applied NEASE to tissue-specific exons reported in VastDB, a resource that provides information on multiple types of AS events detected by RNA-seq from different tissue types and developmental stages [30]. We extracted 2831 exon skipping events and Percent Spliced In values (PSI) from 12 different human tissue types (Additional file 7, see Methods). We then performed hierarchical clustering on the *z* score standardized PSI values (Fig. 2A). The heatmap shows two distinct clusters, where neural-specific and muscle-specific (merged with heart-specific) exons are dominant.

Next, we extracted 56 skipped exons with a high PSI in the muscle tissues and 62 skipped exons with a high PSI in the neural tissues (*z* score ≥ + 2, see the “Methods” section). We checked how many of these events are overlapping with protein features. As shown in Fig. 2B, 27% of the upregulated exons in muscle tissues (13) and 28% of the upregulated exons in the neural tissues (17) overlap with protein features. NEASE also provides statistics of how many of these domains have known binding partners in the joint graph. In the two sets, around 60% of the affected domains have known interactions in our joint graph: 8 binding domains in the muscle tissues and 10 binding domains in the neural tissues (Additional file 2: Tables S4, S5 and Additional file 3: Tables S8, S9). We further identified one affected motif in the gene ATP2B1 in neural exons. For these groups of events, the exact protein complexes involved can be identified, and NEASE statistical analysis can be performed to determine affected pathways. However, it is important to keep in mind that not all affected domains are necessarily interacting domains but could also be regulating gene expression by binding to DNA or RNA [31].

First, we ran a gene set overrepresentation analysis (one-sided hypergeometric test), which we refer to as gene-level enrichment, to detect enriched pathways (see the “Methods” section). Next, we applied NEASE to the same genes to detect pathways affected by AS. Unlike the gene-level enrichment, the results obtained from NEASE in both sets better explain the functional role of the regulated exons (Fig. 2C, D). We also compared with the results from the Network Enrichment Analysis method NEA [32]. NEA is a PPI-based approach that considers all edges for statistical tests. In contrast, NEASE considers only AS-affected edges. For a fair comparison, we run NEA with the same PPI network (BioGRID) and same pathways databases (see the “Methods” section). The results of NEA did not improve over the classic gene-level enrichment (Additional file 1: Figure S2), which suggests that our exon-specific approach helps to narrow down the exact complexes/pathways affected by AS and reduces false positives.

To further validate the robustness of the enrichment obtained by NEASE, we further conducted permutation tests. Here, our null hypothesis is that the tissue-specific exons are not different from a random set of exons in terms of the quality of the functional enrichment (measured as the *p* values of the hypergeometric test). For a more realistic scenario, our background set of exons considers only exon skipping events that can actually be found in these tissues (see the “Methods” section for details). This approach will also help evaluate our methods against known and unknown biases. The empirical *p* values of the permutation test, which indicate the chance of finding an enrichment *p* value as low or lower than the one reported by NEASE, are 0.0008 and 0.0001 for neural and muscles upregulated exons, respectively. These results further demonstrate the robustness of our analysis.

The upregulated exons in heart and muscle tissues were enriched in “Muscle Contraction” pathways (Fig. 2C and Additional file 2: Table S7 ), while, in the gene-level enrichment, the pathways were related to very common subcellular functions such as the Golgi apparatus, which also is an organelle for collecting, modifying or destroying protein products (Fig. 2 C and Additional file 2: Table S6). NEASE provides detailed information about the affected domains and their interaction partners (Additional file 1: Table S1). The domain Tropomyosin (Pfam id: PF00261), which is part of the gene TPM1, e.g., is involved in the regulation of muscle contraction via actin and myosin. GAS2 (Pfam id: PF02187) is a domain of DST, a dystonin encoding gene, which plays a role in maintaining the integrity of the cytoskeleton. AS affects its binding with the gene CALM1 that encodes a calcium-binding protein involved in various calcium-dependent pathways like muscle contraction [33].

**Fig. 3:**
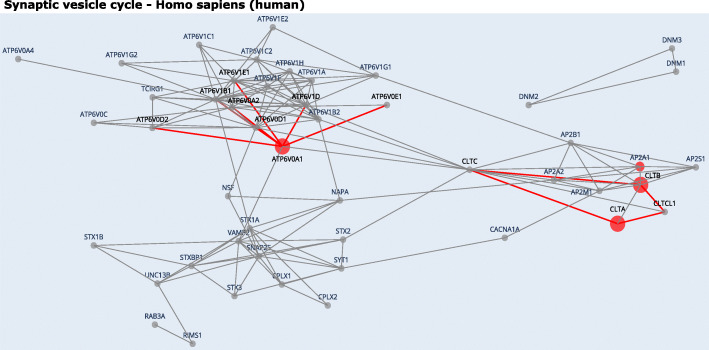
NEASE visually highlights the impact of the AS regulation at the interactome level. The gray nodes represent proteins from the pathway and the red nodes represent genes with AS events. Red edges represent the affected interactions for the nodes with known DDIs, DMIs, or co-resolved structures. The visualization of the pathway “Synaptic vesicle cycle” from the KEGG database for the exons upregulated in the neural tissues shows that the splicing in the genes CLTA and CLTB is co-regulated and affects the interactions of the same complex. Similarly, NEASE highlights the importance of the domain ATP6V0A1 which is upregulated in neural tissues and binds seven proteins from the “Synaptic vesicle cycle” pathway

The exons upregulated in neural tissues showed enrichment in the synaptic vesicle cycle pathway responsible for the communication between neurons (Fig. 2D). Gene-level enrichment performed on par with NEASE, resulting in the same pathway but with a lower rank and significance (adjusted *p* values: 1.494631e−16 using NEASE and 0.0039 using gene-level, Additional file 3: Tables S10 and S11). Notably, NEASE also detected an enrichment in “oxidative phosphorylation”, which is the initiator for powering all major mechanisms mediating brain information processing [34]. The neuron’s energy demands are remarkable both in their intensity and in their dynamic range and quick changes [35–38]. Therefore, AS could modify oxidative phosphorylation to serve tissue-specific needs. Experimental studies have also found that several key enzymes in “oxidative phosphorylation” are spliced, e.g., pyruvate kinase (PKM) that shifts from the PKM2 to the PKM1 isoform [39, 40]. NEASE also provides a detailed view on the affected mechanisms, such as an exon skipping event in the gene ATP6V0A1 overlapping with the V_ATPase_I domain (PFAM id: PF01496) and affecting the binding with seven other proteins from the complex vacuolar ATPase (V-ATPase) (*p* value: 5.853289e−17, Fig. 3, Additional file 1: Table S2 ). V-ATPase is required for synaptic vesicle exocytosis [41] The a1-subunit of the V0 domain in ATP6V0A1 was recently shown to be highly expressed in neurons and to be essential for human brain development [42, 43]. In another example, NEASE identified two co-regulated events of the genes CLTA and CLTB (Fig. 3). CLTA and CLTB genes are involved in Clathrin-dependent endocytosis which forms clathrin-coated vesicles. Both genes play a major role in forming the protein complex of the coated vesicle. Both events affect the same domain Clathrin light chain (Pfam id: PF01086). The Clathrin light chain domain binds to CLTC and CLTCL1 which are the Clathrin heavy chain genes (*p* value: 6.943483e−05). These results suggest that the formation of this complex is co-regulated by AS. A similar finding about the role of the Clathrin light chain in neurons was also described in [44]. NEASE highlights these co-regulated events at the network level (Fig. 3). As a sanity check, we manually checked the PSI values of these critical events identified by NEASE in the Genotype-Tissue Expression data set (GTEx), a comprehensive resource for tissue-specific gene expression and regulation [45]. VastDB includes the quantification of PSI values from 8378 samples (49 tissues and 543 individuals) from GTEx version 6 on their website (https://vastdb.crg.eu/). As shown in the examples in Additional file 1 Figures S4 and S5, the exons are confirmed to be highly upregulated in their respective tissues. The analysis generated from VastDB using NEASE agrees with the latest studies at transcriptomics and proteomics levels that emphasize the crucial role of AS in the function and development of brain and heart tissues [46–48].

### NEASE reveals splicing-related differences of reticulated and mature platelets

AS does not only drive tissue-specific regulation but also plays a major role in cell differentiation and maturation. To illustrate an example of the utility of NEASE in such studies, we used the RNA-seq data set from [49] which compares the transcriptome profiles of reticulated platelets and mature platelets from healthy donors. Reticulated platelets are younger [50], larger in size, and contain more RNA [51]. Moreover, they have a prothrombotic potential and are known to be more abundant in patients with diabetes, acute or chronic coronary syndrome, and in smokers [51–53]. Additionally, elevated levels of reticulated platelets in peripheral blood are predictors of insufficient response to antiplatelet therapies (e.g., aspirin and P2Y12 inhibitors) and are promising novel biomarkers for the prediction of adverse cardiovascular events in different pathological settings [52, 54]. A strong enrichment of pro-thrombotic signaling in reticulated platelets was observed in healthy donors [49]. Comparative transcriptomic analysis revealed a differential expression of several pathways in addition to an enrichment of prothrombotic pathways and transcripts of transmembrane proteins as the collagen receptor GPVI, the thromboxane receptor A2 and the thrombin receptors PAR1 and PAR4. Gene set enrichment analysis indicated an upregulation of entire prothrombotic activation pathways as the thrombin PAR1 and integrin GPIIb/IIIa signaling pathway in reticulated platelets.

**Fig. 4:**
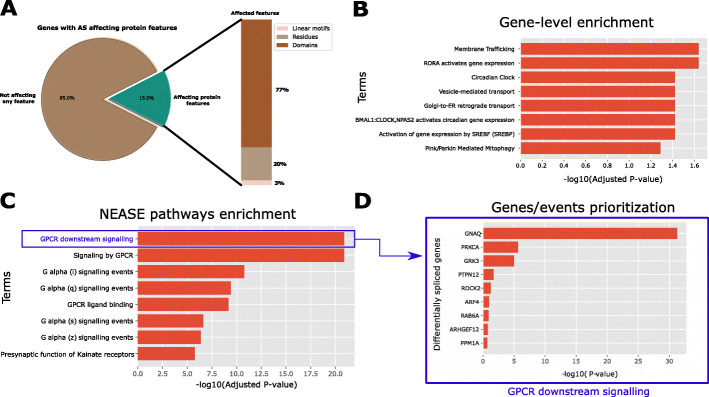
**A** 15 % of differentially spliced exons, between reticulated and mature platelets, are known to encode protein features. For this subset of exons, NEASE enrichment can be performed. **B** Gene level enrichment of all differentially spliced exons in the Reactome database fails to capture the most relevant pathways. **C** In contrast, NEASE shows an enrichment of the GPCR downstream signaling and other related pathways that are well known to be important in platelet activation. **D** A further look at the genes driving the enrichment of the GPCR pathway shows the most relevant genes affected by AS

Since AS has been described to occur in platelets [55], we wanted to investigate the splicing patterns between the previously defined reticulated and mature platelet subgroups. Using MAJIQ [56] (see the “Methods” section), we found 169 differentially spliced genes. From 25 affected protein domains, 17 have known interactions (68% of affected domains, Fig. 4A, Additional file 4: Tables S12 and S13). Other affected protein features include 6 residues involved in PPIs and one linear motif in the gene PAWR.

We observed that the enrichment at the gene-level using the Reactome [57] database ranks general cellular pathways higher, including “Membrane Trafficking” and “Vesicle-mediated transport,” and “Golgi-to-ER retrograde transport.” An exception is the “Circadian Clock” pathway, which is hypothesized to be related to platelet activation [58] (Fig. 4B). The pathway “Platelet activation, signaling and aggregation” was less significant in gene-level enrichment (adjusted *p* value: 0.061, Additional file 4: Table S14) compared to NEASE enrichment (adjusted *p* value: 0.004, Additional file 4: Table S15). Using NEASE, we obtained more meaningful results and unique pathways. As shown in Fig. 4C, the most significant pathways in reticulated platelets are G Protein-Coupled Receptor-related. G proteins are essential in the second phase of platelet-dependent thrombus formation [59]. Furthermore, GPCR isoforms are known to have distinct signaling properties [60]. Other relevant pathways associated with platelet activation are “Hemostasis,” “Thromboxane signaling through tp receptor,” and “Platelet homeostasis.” The full tables for enrichment at the gene level and using NEASE are available in the Additional file 4: Tables S14 and S15. The upregulation of these pathways in reticulated platelets emphasizes their previously described prothrombotic phenotype and their involvement in several downstream signaling processes.

We also looked at the individual AS events driving this enrichment. For each affected feature, NEASE tests if it significantly interacts with the GPCR downstream signaling pathway (Additional file 4: Table S16, see the “Methods” section). Figure 4C illustrates affected genes and their *p* value ranking. The top gene is GNAQ (G-protein subunit alpha q), which is known to be involved in signal transduction in platelets leading to platelet activation [61]. The regulation of the G-protein alpha subunit can be an indication that compared to mature platelets, reticulated platelets are more involved in various signal transduction pathways related to, e.g., pro-thrombotic processes [51]. PRKCA, which also showed different splicing patterns between the two platelet subgroups, plays a major role in the platelet formation process by modulating platelet function [62], megakaryocyte function, and development [63] and negatively regulates pro-platelet formation [64]. Moreover, the regulation of PRKCA binding in reticulated platelets might refer to the young nature of reticulated platelets, which have undergone the pro-platelet formation process more recently than mature platelets [50, 65].

### NEASE characterizes complex disorders such as Multiple Sclerosis

Multiple sclerosis (MS) is a chronic inflammatory demyelinating disease of the central nervous system. Early in the disease course, MS is characterized by focal lesions in the brain induced by an influx of systemic inflammatory cells. These active lesions infiltrated by immune cells and activated microglia are characterized by inflammatory demyelination and axonal loss [66]. The surrounding white matter tissue is termed normal-appearing white matter due to diffuse pathology without focal lesion activity and dense immune activity [67]. The etiology of MS remains unknown. Recently, a systematic literature review found 27 genes that were alternatively spliced in MS patients [68].

We used RNA-Seq of macrodissected areas from postmortem white matter tissue of patients with progressive MS [69]. We compared normal-appearing white matter and active lesions regions from postmortem white matter brains of MS patients. We found 109 differentially spliced genes with 19 affected domains and one linear motif with known interactions, in addition to 6 known interacting residues. In total, NEASE identified 156 affected interactions (Additional file 5: Tables S17 and S18).

Gene-level enrichment ranks high pathways likely irrelevant that are involved in muscle contraction, cardiac conduction, and membrane trafficking, with the exception of Ca2+ ion flow across membranes (Additional file 5: Table S19). Ca2+ is an essential signal molecule for all cell activity. Although deregulation of calcium signaling is related to the pathogenesis of multiple diseases [70], including neurological disorders [71], it is not specific to neuronal tissues. In line with the neurodegenerative and immune-mediated features of MS, NEASE found unique enriched pathways related to brain network signaling and neuronal pathways “Neurotransmitter receptors and postsynaptic signal transmission,” “Transmission across Chemical Synapses,” “Activation of NMDA receptor and postsynaptic events,” “MAPK family signaling cascades,” “Neuronal System”), as well as pathways related to immune responses (“interleukin-17 signaling,” “Toll-Like Receptor 10 (TLF10) Cascade”) (Table 1 and Additional file 5: Table S20). Two other pathways were related to the uptake of anthrax or bacterial toxins. This could be a result of clean-up from toxic inflammatory processes or increased presence of invaders due to the leaky brain-blood-barrier in MS [72–74]. Additionally, it also supports the theory of infections as the trigger of lesion damage in MS [75].

**Table 1:** NEASE enrichment obtained from AS comparison between normal-appearing white matter and acute lesions, from multiple sclerosis patients. The highly enriched pathways belong to Neurotransmitter receptors, MAPK, and bacterial infection. Most of these pathways are hallmarks of MS. The NEASE score is obtained after combining the *p* value with the number of significant genes. The latter is obtained after individual tests for each gene in the column “Spliced genes” (see the “Methods” section)

Pathway name	Spliced genes (number of interactions affecting the pathway)	***p*** value	adj ***p*** value	NEASE score
Neurotransmitter receptors and postsynaptic signal transmission	GRIA1 (7), ATP2B1 (2), BRAF (4), MAP2K4 (1), GRIN1 (4)	4.38e−09	0.000004	16.71
Uptake and function of anthrax toxins	ATP2B1 (1), BRAF (5), MAP2K4 (3)	2.98e−09	0.000004	14.76
Transmission across chemical synapses	GRIA1 (7), ATP2B1 (2), BRAF (4), MAP2K4 (1), GRIN1 (4)	5.65e−08	0.000010	14.49
Uptake and actions of bacterial toxins	ATP2B1 (1), BRAF (5), MAP2K4 (3)	3.46e−08	0.000009	12.92
Neuronal system	GRIA1 (7), ATP2B1 (2), BRAF (4), MAP2K4 (1), GRIN1 (4)	8.71e−07	0.000122	12.11
MAPK family signaling cascades	MYH10 (2), ATP2B1 (1), BRAF (17), MAP2K4 (5), GRIN1 (3)	1.52e−06	0.000184	10.07
Activation of NMDA receptor and postsynaptic events	GRIA1 (2), ATP2B1 (1), BRAF (4), MAP2K4 (1), GRIN1 (3)	2.12e−06	0.000241	9.82
FCERI mediated MAPK activation	MYH10 (1), BRAF (7), MAP2K4 (8)	2.52e−07	0.000038	9.33
RAF/MAP kinase cascade	MYH10 (1), ATP2B1 (1), BRAF (16), MAP2K4 (4), GRIN1 (3)	1.00e−06	0.000130	8.48
Signaling by moderate kinase activity BRAF mutants	MYH10 (1), BRAF (14), MAP2K4 (2)	8.30e−09	0.000004	8.08

As shown in Table 1, the pathway “Uptake and function of anthrax toxins” has the best overall adjusted *p* value, calculated only based on the total number of edges affecting the pathway. When we also included the number of significant genes and calculated NEASE scores (see the “Methods” section), NEASE ranks the pathway “Neurotransmitter receptors and postsynaptic signal transmission” first, and moves pathways such as “Transmission across Chemical Synapses” and “Neuronal System” higher in the rank. These observations illustrate the usefulness of the NEASE score as a complement to the global edge-based enrichment.

Two of the most significant genes in the “Neurotransmitter receptors” pathway were GRIN1 and GRIA1 (Additional file 5: Table S21). GRIN1 encodes GluN1, which is one of the two obligatory subunits for the NMDAR1 receptor, whereas GRIA1 encodes the AMPAR1 subunit. Their ligand is glutamate, and they are both ionotropic receptors and have been associated with MS disease severity [76–78]. Interestingly, AS of MAP2K4 appeared in both brain-related and immune-related pathways, significantly enriched in active lesions *vs* normal-appearing white matter (Table 1). MAP2K4 is a mitogen-activated protein kinase (MAPK) orchestrating multiple biological functions [79, 80]. AS of MAP2K4 has been found in rheumatoid arthritis [81], as well as in pathways of patients with other autoimmune diseases [82]. MS also precedes autoimmune attack, and therefore AS of MAP2K4 in active lesions detected with NEASE may represent dysregulated immune responses originating from the infiltrating immune cells or inflammatory-activated brain cells. This is supported by previous studies that found *(i)* overactivity of MAPK pathways in microglia (the resident immune cell of the brain) during neurodegeneration [83, 84], and *(ii)* increased phosphorylation of MAPK kinases in the systemic immune cells of MS patients [85, 86]. A recent study also characterized activated MS-specific pathways in immune cells from blood using phosphoproteomics. Here, MAP2K4 and its interaction partners (e.g., TAK1) were present in MS-specific signaling activity [87]. Future functional studies on the AS of MAP2K4 may help explain if AS could be the reason for increased phosphorylation and overactivity detected in MS. AS of MAP2K4 could result in switching protein conformation, increasing susceptibility to phosphorylation, or changing the downstream protein cascade.

With NEASE, we were able to specifically detect AS of genes and related pathways already known to be dysregulated within MS from excitotoxicity to inflammation. The detected AS genes in active lesions *vs* normal-appearing white matter demonstrate how major components in signaling activities may be fine-tuned/changed from regulation of a homeostatic state to an inflammatory state. Combining NEASE with functional experiments to understand the biological impact of AS could fuel new therapeutic opportunities for complex neurological diseases as MS. Novel developments in genome-editing tools and gene-specific strategies have made it possible to use antisense oligonucleotides or small modulators for splice modification. This is already used in the rare neuromuscular disease, spinal muscular atrophy, where an antisense oligonucleotide binds to a site near splicing to ensure the inclusion of an exon during the splicing event [78].

### NEASE finds new biomarker candidates for dilated cardiomyopathy

AS might play a role in driving dilated cardiomyopathy (DCM) [88]. DCM is a common heart muscle disease that is often diagnosed with structural abnormalities resulting in impaired contraction. Previous studies have shown a large number of differentially used exons in DCM patients [4, 10]. In this analysis, we used a list of 1212 differentially used exons between DCM patients and controls as reported by Heinig et al. [10]. 29% of these exons overlap with protein features, including 230 domains and 15 linear motifs. (Additional file 6: Tables S22 and S23). In this exon set, both the gene level enrichment and NEASE show very similar results (Additional file 6: Tables S24 and S25). In both methods, we found that the list of exons was enriched in the dilated cardiomyopathy (DCM) pathway from KEGG, as well as, “Adrenergic signaling in cardiomyocytes” and “Regulation of actin cytoskeleton”.

In contrast to gene-level enrichment analysis, NEASE is able to score the contribution of alternatively spliced genes that are interacting with but are not part of the DCM pathway, allowing us to highlight putative biomarkers (Table 2, Additional file 6: Table S26, Additional file 1: Figure S3). The Myosin head domain from the gene MYO19 interacts with 6 other genes associated with DCM: (1) MYL2, which triggers contraction after Ca+ activation [89]; (2–5) TPM1/TPM2/TPM3/TPM4, which encode the TPM protein—the main regulator of muscle contraction [90]; and (6) ACTG, which encodes actin. Interestingly, MYO19 has not been investigated for its role in DCM, while its interacting genes are associated with DCM [91–94]. Additionally, the gene OBSCN has one affected interaction with the TTN gene [95]. The TTN gene itself is also differentially spliced and associated with DCM [95]. OBSCN was recently reported as a new DCM candidate [96, 97]. Another interesting example is CACNA1C (Calcium Voltage-Gated Channel Subunit Alpha1 C), an already known DCM candidate [98]. The differentially spliced exon overlaps with the domain Ion_trans (Pfam id: PF00520) which is essential for myocyte contraction [99]. The affected interaction identified is with the ryanodine receptor 2 (RYR2). In striated muscles, the excitation-contraction coupling is mediated by this complex [100]. Both CACNA1C and RYR2 are part of the KEGG DCM pathway [101]. Alterations in ryanodine receptors were repeatedly reported to be related to heart failure [102–104].

**Table 2:** Enrichment of the pathway “Dilated cardiomyopathy (DCM)” from KEGG for the exons differentially used in DCM patients. The table shows the most significant genes (*p* value< 0.05) (see the “Methods” section)

Differentially spliced genes	DCM associated	Percentage of affected edges associated with DCM	***P*** value	Affected binding (edges) associated with DCM
MYO19	No	6/51	0.000002	MYL2, TPM4, TPM3, TPM2, TPM1, ACTG
OBSCN	No	1/2	0.014	TTN
USP49	No	1/4	0.028	PRKACA
CACNA1C	Yes	1/4	0.028	RYR2

## Discussion

In spite of its importance for biomarker and therapeutic target discovery, differential AS is still not a routine part of transcriptome analysis. A key reason for this could be the lack of suitable methods and software tools for AS-specific functional analysis. Our method NEASE closes this gap and provides a unique view on the impact of AS complementary to functional insights gained from traditional gene-level enrichment analysis. We applied NEASE to four diverse data sets and show that its results generate novel disease-relevant insights and provide valuable context to prior findings on altered RNA- and protein-expression levels consistent with recent literature.

In many cases, NEASE improves over gene-level enrichment analysis focusing on differentially spliced genes. One potential reason for this could be that not all AS events are necessarily functional [11, 12]. NEASE mitigates this by focusing on AS events that affect protein domains. However, it is important to keep in mind that this is not the only way to define functional AS events. AS also affects interacting disordered regions [14] or facilitates nonsense-mediated decay [105].

AS events could also lead to completely different functions or interactions [106], e.g., two isoforms can have different interaction partners depending on the inclusion or loss of a single domain [13]. Such changes in the interactome can not be captured with gene-level enrichment which has a strict focus on nodes rather than edges. With NEASE, we could show that integrating structural information at the exon level and PPI networks helps to identify the functional impact of differentially spliced and co-regulated exons. In practice, we consider both approaches as complementary and recommend running gene-level and edge-level enrichments together (both supported by the NEASE package). Note that while our analysis focuses on exon skipping events as the most studied event type, our method is generally agnostic to the event type.

NEASE relies on structurally annotated interactions and existing pathway annotations from databases such as KEGG [101] and Reactome [57]. Leveraging reliable structural information and established pathways likely removes many false positive PPI from considerations. While the structural annotations are generally of high quality, it should be noted that their coverage is still limited and, thus, the number of exons considered in our method is comparably low. For instance, the percentage of considered exons, in our example datasets, ranges between 15 and 30%, which is still far from being a global analysis of AS. Expanding the annotations at isoform-level and more widespread availability of structural information will greatly raise the usefulness of NEASE in the future. We also emphasize that while all events can potentially affect protein interactions on the domain level, not all AS events yield functional isoforms and other processes such as nonsense-mediated decay need to be considered as well. In the future, further progress is urgently needed to link transcriptomics and proteomics for better characterization and understanding of the exact impact of AS events. With our current approach, a large fraction of the PPI network remains unexplored, suggesting that adapting de novo network enrichment methods such as KeyPathwayMiner [107] towards AS could be a promising research direction to uncover previously unknown disease mechanisms. NEASE currently considers the immediate neighborhood of a pathway in the PPI network. When carefully considering the expected increase in false positives, one could also increase the size of the pathway neighborhood using, e.g., a fixed radius for shortest paths. While these are attractive approaches, the biases of the PPI towards hubs, as well as the high number of false (or missing) edges of PPI, in its current form, make such approaches hard to control and statistically challenging. Even though NEASE is relatively conservative, we demonstrated that it is simple, robust, and generates meaningful and interpretable results. Thus, it provides an unprecedented opportunity to understand the functional impact of tissue-, developmental- and disease-specific AS in a system biology manner.

## Conclusions

While a plethora of gene set enrichment methods have been proposed in recent years, AS is typically not addressed specifically. Thus, NEASE closes an important gap in functional enrichment analysis of transcriptomics data. The analyses described here, confirm the widespread impact of AS in multiple biological processes and disorders. In the future, we plan to extend NEASE with further model organisms and to add structural annotations covering more types of AS events. Finally, we plan to integrate NEASE with the DIGGER web tool [21] for seamless downstream analysis of AS in the web browser with the vision of establishing functional AS event analysis as a routine step in the transcriptomic analysis.

## Methods

### NEASE data sources

We construct a human structurally annotated PPI as described previously [21]. Briefly, we integrate DDI and PPI information into a joint network where DDIs were obtained from 3did (v2019_01 [26]) and DOMINE (v2.0 [25] including high- and mid-confidence interactions) and PPIs were obtained from BioGRID 3.5 [24]. In summary, out of 410,961 interactions from the human interactome 52,467 have at least one domain interaction. The linear motif instances and their interactions were downloaded from the ELM website and mapped to the respective exons. We found 3926 PPIs that are confirmed by at least one source of DMI. Position-specific PPI based on experimentally resolved structure from the PDB was obtained from [21]. In total, 16,161 PPIs were enriched by at least one residue-level interaction. From the combination of all these resources the final structurally annotated graph contained in total 60,235 interactions. Each one of these interactions is annotated with one or multiple levels of evidence (DDIs, DMI, residues). The mapping of exons to their protein features was performed using the Biomart mapping table, Pfam, and ELM annotations [22, 23, 108]. We obtain the biological pathways with their gene list from KEGG [57] and Reactome [101] integrated into the ConsensusPathDB database [28].

### Statistical tests and pathway scores

Gene-level enrichment is performed using a hypergeometric test from the package GSEAPY (a Python wrapper for Enrichr [109]) by considering all genes with (differential) AS events. Network enrichment analysis at the gene level was performed using the EviNet web server (www.evinet.org), which is an implementation of the randomization algorithm NEA [32]. To achieve a fair comparison with NEASE, we run NEA using BioGRID as a PPI database and Reactome and KEGG as pathways references to match the exact conditions of the NEASE analysis.

For NEASE enrichment, we filtered the PPI graph *G*=(*V*, *E*), where *V* is the set of genes and *E* is the set of edges, to a subgraph *G*′=(*V*′, *E*′) containing only structurally annotated interactions *E*′ and their nodes *V*′. An interaction is considered structurally annotated if it is supported by at least one of these resources: domain-domain interactions, motif-domain interactions, or residue-level interactions. For a submitted query list of exons, NEASE first identifies affected domains, linear motifs, and residues that overlap with the exons and their interactions. Let *N* be two times the number of edges in *G*′ (the degree of the network) and n be the number of affected edges from the query. These edges are then considered using a test modified from [110]. For every pathway *P* with degree *K*, let *k* be the number of affected edges that are connected to *P*. We model *X* whose outcome is *k* as a random variable following a hypergeometric distribution:

$$ \mathrm{X}\sim \mathrm{Hypergeometric}\left(\mathrm{n}=\mathrm{number}\ \mathrm{of}\ \mathrm{affected}\ \mathrm{edges},\mathrm{K}=\mathrm{degree}\ \mathrm{of}\ \mathrm{P},\mathrm{N}=\mathrm{degree}\ \mathrm{of}\ \mathrm{G}\hbox{'}\right) $$

where *k* is considered as the number of observed successes out of *n* draws, from a population of size *N* containing *K* success. Subsequently, NEASE tests if the number *k* is significant using a one-sided hypergeometric test (over-representation). In contrast to the test proposed in [110], our test only includes structurally annotated edges and the ones likely to be impacted by AS in order to improve the signal-to-noise ratio. For illustration purposes, in the example of Fig. 1B, the overall number of affected edges by AS is *n*=7, and *K*=11 is the total degree of pathway *A* (11 possible success), the number of affected edges that are linked to the pathway is *k*=4. The enrichment *p* value of pathway A corresponds then to the significance of this last number. After testing for multiple pathways, the obtained *p* values for the edge-level enrichment are corrected, using the Benjamini-Hochberg method [111]. The detailed pseudocode of this algorithm is explained in Additional file 1: Figure S6, Algorithm 1.

For a pathway of interest, a similar test can be applied to determine if a splicing event significantly affects interactions of a specific gene with this pathway (Additional file 1: Figure S6, Algorithm 2 and Fig. 1B, C). Here, *n* is the number of all affected interactions (edges) of a spliced gene and *k* is the number of affected interactions (edges) across genes that are linked to the pathway of interest. In the example of Fig. 1B, C, the gene G2 can be connected to pathway *A* just by chance due to its high number of affected interactions. For this reason, it is ranked lower than the genes G3 and G4.

As a result, for every pathway, NEASE provides an overall *p* value, as well as the most significant genes. Since the *p* value only depends on the overall number of affected edges but not on the number of genes, the *p* value can be heavily influenced by hub genes. To reduce this influence, an optional score (NEASE Score, Eq.: 1) can be computed by NEASE to scale the natural logarithm of the *p* value with the total number of significant genes using a cutoff from the user (for instance *p* value ≤ 0.05):

1 $$ \mathrm{NEASE}\ \mathrm{score}=-\sqrt{g}\times {\log}_{10}\left(p\;\mathrm{value}\right) $$

where *g* is the total number of significantly connected genes obtained after testing individual spliced genes. Thus, the NEASE Score prioritizes pathways that are affected by a larger number of spliced genes rather than pathways that have a larger number of affected interactions (edges). The user can choose to rank enrichment based on the adjusted *p* value or by the NEASE score.

For permutation tests, the set of highly confident 2831 exon skipping events obtained after the initial quality filtering is considered as the background set of exons. We compared the enrichment obtained in the pathways “Muscle Contraction” and “Synaptic vesicle cycle” from the set of exons upregulated in muscles/heart and neural tissues respectively, with 10,000 random sets of exons of the same size and then derived distribution of *p* values. The empirical *p* values were then obtained by asking how likely it is to obtain a *p* value as low or more extreme than the one reported by NEASE in the original set of neural and muscles upregulated exons.

### VastDB events processing

PSI values of the exon skipping events from VastDB were quantified by the developers using vast tools [30, 112]. In our analysis, we extracted the PSI values for 32 experiments belonging to 12 main tissues: muscles/heart, neural (whole brain, cortex, and peripheral retina), placental, epithelial, digestive (colon and stomach), liver, kidney, adipose, testis, immune-hematopoietic, and ovary. We then filtered out the events with low read coverage (VLOW) and performed hierarchical clustering of standardized values (*z* scores). For every exon, we calculated the mean of PSI values from the samples of the same tissues. To extract muscles/heart and neural-specific exons and to ensure that we only consider functional events, we applied two filters: namely that the exon PSI value in the relevant tissue is higher than 20 and that the *z* score is higher than 2.

### RNA-Seq analysis

Raw RNA-Seq reads for two types of platelets and multiple sclerosis patients were downloaded from the GEO repository (access numbers: GSE126448 and GSE138614 ). The number of samples and sequencing depth are reported in Additional file 1: Table S3. RNA-Seq reads were aligned to the reference human genome (hg38) using STAR 2.7 [113] in a 2-pass mode and filtered for uniquely mapped reads. Differential AS analysis was performed by MAJIQ [56] with default parameters, and with a threshold of P(dPSI > 20%) > 0.95.

### NEASE: The Python package

NEASE’s Python package relies on NumPy [114], pandas [115], NetworkX [116], SciPy [117], and Statsmodels [118]. The gene-level enrichment is also supported in the NEASE package using the Python implementation of Enrichr [109]. To speed up the edge hypergeometric test, the total degree of every pathway in the structural PPI, as well as the overall degree of the network were pre-computed. For visualization, we use the complete PPI (not the structural PPI) and extract connected subnetworks from each pathway as well as spliced genes and their interactions with the extracted modules. The position of nodes is computed using the Fruchterman-Reingold force-directed algorithm implemented in NetworkX [119]. The interactive visualization for individual genes and events is implemented with information from the DIGGER database and the Plotly package.

The package provides the option to automatically filter exons that are likely to disturb the open reading frame of the transcript based on the prediction in [30]. In the case of multiple AS events affecting the same genes, we consider every event individually and identify all protein features. The standard input of the package is a DataFrame object with the exon coordinates and Ensembl IDs of the genes. The package also supports the output of multiple AS differential detection tools such as rMATs [120], Whippet [121], and also tools that are event-based such as MAJIQ [56] where NEASE only considers annotated exons. NEASE is released as open-source under the GPLv3 license and is available at (https://github.com/louadi/NEASE). Step-by-step tutorials for running NEASE are available at (https://github.com/louadi/NEASE-tutorials).

## Supplementary Information

**Additional file 1:** **Tables S1-S3.** **Table S1.** Enrichment of the pathway “Muscle contraction” from Reactome for the exons upregulated in the muscles generated by the NEASE package. **Table S2.** Enrichment of the pathway “Synaptic vesicle cycle” from KEGG for the exons upregulated in the neural tissues generated by the NEASE package. **Table S3.** RNA-Seq samples used in the study. **Figs.** **S1-S6.** **Fig. S1.** Node degree distribution of the classic PPI and structurally annotated PPI, the latter contains only interactions with evidence from DDIs and DMIs or residue-level evidence from the co-resolved structure. **Fig S2.** Network Enrichment Analysis using EviNet webtool for exons upregulated in muscles and neural tissues. **Fig. S3.** NEASE visualization highlights the interactions of differentially spliced genes with the DCM pathway. **Fig. S4.** The PSI values of two exon skipping events in the genes TPM1 and DST from the GTEx dataset confirm that both the exons are upregulated in muscles and heart tissues. **Fig. S5.** The PSI values of two exon skipping events in the genes CLTA and CLTB from the GTEx dataset confirm that both the exons are upregulated in neural tissues. **Fig. S6.** Pseudocode of NEASE algorithm.

**Additional file 2:** **Tables S4-S7.** The analysis of the upregulated exons in muscles. **Table S4:** List of spliced domains. **Table S5:** List of affected edges. **Table S6:** Gene-level enrichment. **Table S7:** NEASE enrichment.

**Additional file 3:** **Tables S8-S11.** The analysis of the upregulated exons in neural. **Table S8.** List of spliced domains. **Table S9.** List of affected edges. **Table S10.** Gene-level enrichment. **Table S11.** NEASE enrichment.

**Additional file 4:** **Tables S12-S16.** Differential splicing analysis between reticulated platelets and mature platelets. **Table S12.** List of spliced domains. **Table S13.** List of affected edges. **Table S14.** Gene-level enrichment. **Table S15.** NEASE enrichment. **Table S16.** Enrichment of the pathway “GPCR downstream signal”.

**Additional file 5:** **Tables S17-S21.** Differential splicing analysis between normal-appearing white matter and acute lesion from multiple sclerosis patients. **Table S17.** List of spliced domains. **Table S18.** List of affected edges. **Table S19.** Gene-level enrichment. **Table S20.** NEASE enrichment. **Table S21.** Enrichment of the pathway “Neurotransmitter receptors and postsynaptic signal transmission”.

**Additional file 6:** **Tables S22-S24.** Differential splicing analysis between Dilated Cardiomyopathy patients and controls. **Table S22.** List of spliced domains. **Table S23.** List of affected edges. **Table S24.** Gene-level enrichment. **Table S25.** NEASE enrichment. **Table S26.** Enrichment of the pathway “Dilated cardiomyopathy”.

**Additional file 7:** **Table S27.** The PSI values for VastDB exons in different tissues.

**Additional file 8.** Review history.

## Data Availability

RNA sequencing data for reticulated platelets was provided by the authors [49] and it is freely available at GEO (access number: GSE126448). Multiple sclerosis Raw sequence was provided by the authors [69] and freely available at GEO (access number: GSE138614). Dilated Cardiomyopathy raw data is available in the European Genome-phenome Archive (Dataset ID: EGAS00001002454), in our analysis, we used pre-processed data from the manuscript [10]. VastDB dataset for humans (hg19) was downloaded from https://vastdb.crg.eu/wiki/Downloads. The linear motifs instances and interactions were downloaded from (http://elm.eu.org/). The generated joint graphs and the exon mapping databases are available on the DIGGER database website https://exbio.wzw.tum.de/digger/download. NEASE is released as open-source under the GPLv3 license and is available at GitHub [122] and deposited to Zenodo [123]. All processed datasets, as well as step-by-step tutorials for using NEASE to reproduce the results presented in this paper, are available at https://github.com/louadi/NEASE-tutorials and deposited to Zenodo [124].

## References

[1] Stamm S, Ben-Ari S, Rafalska I, Tang Y, Zhang Z, Toiber D, Thanaraj TA, Soreq H (2005). Function of alternative splicing. Gene.

[2] Yeo G, Holste D, Kreiman G, Burge CB (2004). Variation in alternative splicing across human tissues. Genome Biol.

[3] Baralle F, Giudice J. Alternative splicing as a regulator of development and tissue identity. Nat Rev Mol Cell Biol. 2017;18(7) Available from:. 10.1038/nrm.2017.27. 10.1038/nrm.2017.27 PMC6839889 28488700

[4] Beqqali A (2018). Alternative splicing in cardiomyopathy. Biophys Rev.

[5] Douglas AGL, Wood MJA (2013). Splicing therapy for neuromuscular disease. Mol Cell Neurosci.

[6] ​​Evsyukova I, Somarelli JA, Gregory SG, Garcia-Blanco MA. Alternative splicing in multiple sclerosis and other 673 autoimmune diseases. RNA Biology. 2010;7(4)462–73. 10.4161/rna.7.4.12301. 10.4161/rna.7.4.12301 PMC3070910 20639696

[7] López-Bigas N, Audit B, Ouzounis C, Parra G, Guigó R (2005). Are splicing mutations the most frequent cause of hereditary disease?. FEBS Lett.

[8] Karlebach G, Veiga DFT, Mays AD, Chatzipantsiou C, Barja PP, Chatzou M, et al. The impact of biological sex on alternative splicing. bioRxiv. 2020:490904. 10.1101/490904.

[9] Tollervey JR, Wang Z, Hortobágyi T, Witten JT, Zarnack K, Kayikci M (2011). Analysis of alternative splicing associated with aging and neurodegeneration in the human brain. Genome Res.

[10] Heinig M, Adriaens ME, Schafer S, van Deutekom HWM, Lodder EM, Ware JS, Schneider V, Felkin LE, Creemers EE, Meder B, Katus HA, Rühle F, Stoll M, Cambien F, Villard E, Charron P, Varro A, Bishopric NH, George AL, dos Remedios C, Moreno-Moral A, Pesce F, Bauerfeind A, Rüschendorf F, Rintisch C, Petretto E, Barton PJ, Cook SA, Pinto YM, Bezzina CR, Hubner N (2017). Natural genetic variation of the cardiac transcriptome in non-diseased donors and patients with dilated cardiomyopathy. Genome Biol.

[11] Tress ML, Abascal F, Valencia A (2017). Alternative Splicing May Not Be the Key to Proteome Complexity. Trends Biochem Sci. Elsevier Ltd.

[12] Melamud E, Moult J (2009). Stochastic noise in splicing machinery. Nucleic Acids Res.

[13] Yang X, Coulombe-Huntington J, Kang S, Sheynkman GM, Hao T, Richardson A, Sun S, Yang F, Shen YA, Murray RR, Spirohn K, Begg BE, Duran-Frigola M, MacWilliams A, Pevzner SJ, Zhong Q, Trigg SA, Tam S, Ghamsari L, Sahni N, Yi S, Rodriguez MD, Balcha D, Tan G, Costanzo M, Andrews B, Boone C, Zhou XJ, Salehi-Ashtiani K, Charloteaux B, Chen AA, Calderwood MA, Aloy P, Roth FP, Hill DE, Iakoucheva LM, Xia Y, Vidal M (2016). Widespread Expansion of Protein Interaction Capabilities by Alternative Splicing. Cell.

[14] Buljan M, Chalancon G, Eustermann S, Wagner GP, Fuxreiter M, Bateman A (2012). Tissue-specific splicing of disordered segments that embed binding motifs rewires protein interaction networks. Mol Cell.

[15] da Costa PJ, Menezes J, Romão L (2017). The role of alternative splicing coupled to nonsense-mediated mRNA decay in human disease. Int J Biochem Cell Biol.

[16] Kristoffer V-S (2017). Sandelin A.

[17] delafuente L, Arzalluz-luque Á, Tardáguila M, Delrisco H, Martí C, Tarazona S, et al. tappAS: a comprehensive computational framework for the analysis of the functional impact of differential splicing. Genome Biol. 2020;21(1) Available from:. 10.1186/s13059-020-02028-w. 10.1186/s13059-020-02028-w PMC7236505 32423416

[18] Gal-Oz ST, Haiat N, Eliyahu D, Shani G, Shay T (2021). DoChaP: the domain change presenter. Nucleic Acids Res.

[19] Ctor Climente-Gonzá Lez H, Porta-Pardo E, Godzik A, Correspondence EE, Eyras E (2017). The Functional Impact of Alternative Splicing in Cancer. Cell Rep.

[20] Tranchevent L-C, Aubé F, Dulaurier L, Benoit-Pilven C, Rey A, Poret A (2017). Identification of protein features encoded by alternative exons using Exon Ontology. Genome Res.

[21] Louadi Z, Yuan K, Gress A, Tsoy O, Kalinina OV, Baumbach J, et al. DIGGER: exploring the functional role of alternative 709 splicing in protein interactions. Nucleic Acids Res. 2020;49(D1):D309-D318. 10.1093/nar/gkaa768. 10.1093/nar/gkaa768 PMC7778957 32976589

[22] Kumar M, Gouw M, Michael S. Amano-S ´ Anchez HS´, Pancsa R, Glavina J, et al. ELM-the eukaryotic linear motif resource in 2020. Nucleic Acids Res. 2020;48. Available from: https://academic.oup.com/nar/article/48/D1/D296/5611669. 10.1093/nar/gkz1030. 10.1093/nar/gkz1030 PMC7145657 31680160

[23] El-Gebali S, Mistry J, Bateman A, Eddy SR, Luciani A, Potter SC (2019). The Pfam protein families database in 2019. Nucleic Acids Res.

[24] Oughtred R, Stark C, Breitkreutz B-J, Rust J, Boucher L, Chang C, Kolas N, O’Donnell L, Leung G, McAdam R, Zhang F, Dolma S, Willems A, Coulombe-Huntington J, Chatr-aryamontri A, Dolinski K, Tyers M (2019). The BioGRID interaction database: 2019 update. Nucleic Acids Res.

[25] Yellaboina S, Tasneem A, Zaykin DV, Raghavachari B, Jothi R (2011). DOMINE: a comprehensive collection of known and predicted domain-domain interactions. Nucleic Acids Res.

[26] Mosca R, Céol A, Stein A, Olivella R, Aloy P (2014). 3did: a catalog of domain-based interactions of known three-dimensional structure. Nucleic Acids Res.

[27] Berman HM, Westbrook J, Feng Z, Gilliland G, Bhat TN, Weissig H, Shindyalov IN, Bourne PE (2000). The Protein Data Bank. Nucleic Acids Res.

[28] Kamburov A, Stelzl U, Lehrach H, Herwig R (2013). The ConsensusPathDB interaction database: 2013 update. Nucleic Acids Res.

[29] Ellis JD, Barrios-Rodiles M, Çolak R, Irimia M, Kim T, Calarco JA (2012). Tissue-Specific Alternative Splicing Remodels Protein-Protein Interaction Networks. Mol Cell.

[30] Tapial J, Ha KCH, Sterne-Weiler T, Gohr A, Braunschweig U, Hermoso-Pulido A (2017). An atlas of alternative splicing profiles and functional associations reveals new regulatory programs and genes that simultaneously express multiple major isoforms. Genome Res.

[31] Seo PJ, Kim MJ, Ryu J-Y, Jeong E-Y, Park C-M (2011). Two splice variants of the IDD14 transcription factor competitively form nonfunctional heterodimers which may regulate starch metabolism. Nat Commun.

[32] Alexeyenko A, Lee W, Pernemalm M, Guegan J, Dessen P, Lazar V, Lehtiö J, Pawitan Y (2012). Network enrichment analysis: extension of gene-set enrichment analysis to gene networks. BMC Bioinformatics.

[33] Tansey MG, Luby-Phelps K, Kamm KE, Stull JT (1994). Ca(2+)-dependent phosphorylation of myosin light chain kinase decreases the Ca2+ sensitivity of light chain phosphorylation within smooth muscle cells. J Biol Chem.

[34] Hall CN, Klein-Flügge MC, Howarth C, Attwell D (2012). Oxidative phosphorylation, not glycolysis, powers presynaptic and postsynaptic mechanisms underlying brain information processing. J Neurosci.

[35] Sanganahalli BG, Herman P, Blumenfeld H, Hyder F (2009). Oxidative neuroenergetics in event-related paradigms. J Neurosci.

[36] Vergara RC, Jaramillo-Riveri S, Luarte A, Moënne-Loccoz C, Fuentes R, Couve A, Maldonado PE (2019). The Energy Homeostasis Principle: Neuronal Energy Regulation Drives Local Network Dynamics Generating Behavior. Front Comput Neurosci.

[37] Du F, Zhu X-H, Zhang Y, Friedman M, Zhang N, Ugurbil K (2008). Tightly coupled brain activity and cerebral ATP metabolic rate. Proc Natl Acad Sci U S A.

[38] Howarth C, Gleeson P, Attwell D (2012). Updated energy budgets for neural computation in the neocortex and cerebellum. J Cereb Blood Flow Metab.

[39] Magistretti PJ, Allaman I (2015). A cellular perspective on brain energy metabolism and functional imaging. Neuron.

[40] Zheng X, Boyer L, Jin M, Mertens J, Kim Y, Ma L (2016). Metabolic reprogramming during neuronal differentiation from aerobic glycolysis to neuronal oxidative phosphorylation. Elife.

[41] Hiesinger PR, Fayyazuddin A, Mehta SQ, Rosenmund T, Schulze KL, Zhai RG, Verstreken P, Cao Y, Zhou Y, Kunz J, Bellen HJ (2005). The v-ATPase V0 subunit a1 is required for a late step in synaptic vesicle exocytosis in Drosophila. Cell.

[42] Aoto K, Kato M, Akita T, Nakashima M, Mutoh H, Akasaka N, Tohyama J, Nomura Y, Hoshino K, Ago Y, Tanaka R, Epstein O, Ben-Haim R, Heyman E, Miyazaki T, Belal H, Takabayashi S, Ohba C, Takata A, Mizuguchi T, Miyatake S, Miyake N, Fukuda A, Matsumoto N, Saitsu H (2021). ATP6V0A1 encoding the a1-subunit of the V0 domain of vacuolar H+-ATPases is essential for brain development in humans and mice. Nat Commun.

[43] Poëa-Guyon S, Amar M, Fossier P, Morel N (2006). Alternative splicing controls neuronal expression of v-ATPase subunit a1 and sorting to nerve terminals. J Biol Chem.

[44] Redlingshöfer L, McLeod F, Chen Y, Camus MD, Burden JJ, Palomer E (2020). Clathrin light chain diversity regulates membrane deformation in vitro and synaptic vesicle formation in vivo. Proc Natl Acad Sci U S A.

[45] Lonsdale J, Thomas J, Salvatore M, Phillips R, Lo E, Shad S, Hasz R, Walters G, Garcia F, Young N, Foster B, Moser M, Karasik E, Gillard B, Ramsey K, Sullivan S, Bridge J, Magazine H, Syron J, Fleming J, Siminoff L, Traino H, Mosavel M, Barker L, Jewell S, Rohrer D, Maxim D, Filkins D, Harbach P, Cortadillo E, Berghuis B, Turner L, Hudson E, Feenstra K, Sobin L, Robb J, Branton P, Korzeniewski G, Shive C, Tabor D, Qi L, Groch K, Nampally S, Buia S, Zimmerman A, Smith A, Burges R, Robinson K, Valentino K, Bradbury D, Cosentino M, Diaz-Mayoral N, Kennedy M, Engel T, Williams P, Erickson K, Ardlie K, Winckler W, Getz G, DeLuca D, MacArthur D, Kellis M, Thomson A, Young T, Gelfand E, Donovan M, Meng Y, Grant G, Mash D, Marcus Y, Basile M, Liu J, Zhu J, Tu Z, Cox NJ, Nicolae DL, Gamazon ER, Im HK, Konkashbaev A, Pritchard J, Stevens M, Flutre T, Wen X, Dermitzakis ET, Lappalainen T, Guigo R, Monlong J, Sammeth M, Koller D, Battle A, Mostafavi S, McCarthy M, Rivas M, Maller J, Rusyn I, Nobel A, Wright F, Shabalin A, Feolo M, Sharopova N, Sturcke A, Paschal J, Anderson JM, Wilder EL, Derr LK, Green ED, Struewing JP, Temple G, Volpi S, Boyer JT, Thomson EJ, Guyer MS, Ng C, Abdallah A, Colantuoni D, Insel TR, Koester SE, Little AR, Bender PK, Lehner T, Yao Y, Compton CC, Vaught JB, Sawyer S, Lockhart NC, Demchok J, Moore HF (2013). The Genotype-Tissue Expression (GTEx) project. Nat Genet.

[46] Rodriguez JM, Pozo F, di Domenico T, Vazquez J, Tress ML (2020). An analysis of tissue-specific alternative splicing at the protein level. Orengo CA, editor. PLoS Comput Biol.

[47] Raj B, Blencowe BJ (2015). Alternative Splicing in the Mammalian Nervous System: Recent Insights into Mechanisms and Functional Roles. Neuron.

[48] Su C-H, Dhananjaya D, Tarn W-Y (2018). Alternative Splicing in Neurogenesis and Brain Development. Front Mol Biosci.

[49] Bongiovanni D, Santamaria G, Klug M, Santovito D, Felicetta A, Hristov M, von Scheidt M, Aslani M, Cibella J, Weber C, Moretti A, Laugwitz KL, Peano C, Bernlochner I (2019). Transcriptome Analysis of Reticulated Platelets Reveals a Prothrombotic Profile. Thromb Haemost.

[50] Ault KA, Knowles C (1995). In vivo biotinylation demonstrates that reticulated platelets are the youngest platelets in circulation. Exp Hematol.

[51] Karpatkin S (1969). Heterogeneity of human platelets. II. Functional evidence suggestive of young and old platelets. J Clin Invest.

[52] Cesari F, Marcucci R, Gori AM, Caporale R, Fanelli A, Casola G, Balzi D, Barchielli A, Valente S, Giglioli C, Gensini G, Abbate R (2013). Reticulated platelets predict cardiovascular death in acute coronary syndrome patients. Thromb Haemost.

[53] Guthikonda S, Alviar CL, Vaduganathan M, Arikan M, Tellez A, DeLao T, Granada JF, Dong JF, Kleiman NS, Lev EI (2008). Role of reticulated platelets and platelet size heterogeneity on platelet activity after dual antiplatelet therapy with aspirin and clopidogrel in patients with stable coronary artery disease. J Am Coll Cardiol.

[54] Muronoi T, Koyama K, Nunomiya S, Lefor AK, Wada M, Koinuma T, Shima J, Suzukawa M (2016). Immature platelet fraction predicts coagulopathy-related platelet consumption and mortality in patients with sepsis. Thromb Res.

[55] Nassa G, Giurato G, Cimmino G, Rizzo F, Ravo M, Salvati A, Nyman TA, Zhu Y, Vesterlund M, Lehtiö J, Golino P, Weisz A, Tarallo R (2018). Splicing of platelet resident pre-mRNAs upon activation by physiological stimuli results in functionally relevant proteome modifications. Sci Rep.

[56] Vaquero-Garcia J, Barrera A, Gazzara MR, González-Vallinas J, Lahens NF, Hogenesch JB (2016). A new view of transcriptome complexity and regulation through the lens of local splicing variations. Elife.

[57] Fabregat A, Jupe S, Matthews L, Sidiropoulos K, Gillespie M, Garapati P, Haw R, Jassal B, Korninger F, May B, Milacic M, Roca CD, Rothfels K, Sevilla C, Shamovsky V, Shorser S, Varusai T, Viteri G, Weiser J, Wu G, Stein L, Hermjakob H, D’Eustachio P (2018). The Reactome Pathway Knowledgebase. Nucleic Acids Res.

[58] Scheer FAJL, Michelson AD, Frelinger AL, Evoniuk H, Kelly EE, McCarthy M (2011). The human endogenous circadian system causes greatest platelet activation during the biological morning independent of behaviors. PLoS One.

[59] Offermanns S (2006). Activation of Platelet Function Through G Protein–Coupled Receptors. Circ Res.

[60] Marti-Solano M, Crilly SE, Malinverni D, Munk C, Harris M, Pearce A, Quon T, Mackenzie AE, Wang X, Peng J, Tobin AB, Ladds G, Milligan G, Gloriam DE, Puthenveedu MA, Babu MM (2020). Combinatorial expression of GPCR isoforms affects signalling and drug responses. Nature.

[61] Jalagadugula G, Dhanasekaran DN, Kim S, Kunapuli SP, Rao AK (2006). Early growth response transcription factor EGR-1 regulates Galphaq gene in megakaryocytic cells. J Thromb Haemost.

[62] Moore SF, van den Bosch MTJ, Hunter RW, Sakamoto K, Poole AW, Hers I (2013). Dual regulation of glycogen synthase kinase 3 (GSK3)α/β by protein kinase C (PKC)α and Akt promotes thrombin-mediated integrin αIIbβ3 activation and granule secretion in platelets. J Biol Chem.

[63] Harper MT, Poole AW (2010). Diverse functions of protein kinase C isoforms in platelet activation and thrombus formation. J Thromb Haemost.

[64] Williams CM, Harper MT, Poole AW (2014). PKCα negatively regulates in vitro proplatelet formation and in vivo platelet production in mice. Platelets.

[65] Ault KA, Rinder HM, Mitchell J, Carmody MB, Vary CP, Hillman RS (1992). The significance of platelets with increased RNA content (reticulated platelets). A measure of the rate of thrombopoiesis. Am J Clin Pathol.

[66] Bö L, Dawson TM, Wesselingh S, Mörk S, Choi S, Kong PA (1994). Induction of nitric oxide synthase in demyelinating regions of multiple sclerosis brains. Ann Neurol.

[67] Ludwin SK (2006). The pathogenesis of multiple sclerosis: relating human pathology to experimental studies. J Neuropathol Exp Neurol.

[68] Hecker M, Rüge A, Putscher E, Boxberger N, Rommer PS, Fitzner B, Zettl UK (2019). Aberrant expression of alternative splicing variants in multiple sclerosis - A systematic review. Autoimmun Rev.

[69] Elkjaer ML, Frisch T, Reynolds R, Kacprowski T, Burton M, Kruse TA, Thomassen M, Baumbach J, Illes Z (2019). Molecular signature of different lesion types in the brain white matter of patients with progressive multiple sclerosis. Acta Neuropathol Commun.

[70] Gissel H (2000). Ca2+ accumulation and cell damage in skeletal muscle during low frequency stimulation. Eur J Appl Physiol.

[71] Maléth J, Hegyi P (2016). Ca2+ toxicity and mitochondrial damage in acute pancreatitis: translational overview. Philos Trans R Soc Lond B Biol Sci.

[72] Minagar A, Alexander JS (2003). Blood-brain barrier disruption in multiple sclerosis. Mult Scler.

[73] Claudio L, Raine CS, Brosnan CF (1995). Evidence of persistent blood-brain barrier abnormalities in chronic-progressive multiple sclerosis. Acta Neuropathol.

[74] Ortiz GG, Pacheco-Moisés FP, Macías-Islas MÁ, Flores-Alvarado LJ, Mireles-Ramírez MA, González-Renovato ED (2014). Role of the blood-brain barrier in multiple sclerosis. Arch Med Res.

[75] Ascherio A, Munger KL (2007). Environmental risk factors for multiple sclerosis. Part I: the role of infection. Ann Neurol.

[76] Baranzini SE, Srinivasan R, Khankhanian P, Okuda DT, Nelson SJ, Matthews PM, Hauser SL, Oksenberg JR, Pelletier D (2010). Genetic variation influences glutamate concentrations in brains of patients with multiple sclerosis. Brain.

[77] Strijbis EMM, Inkster B, Vounou M, Naegelin Y, Kappos L, Radue E-W, Matthews PM, Uitdehaag BMJ, Barkhof F, Polman CH, Montana G, Geurts JJG (2013). Glutamate gene polymorphisms predict brain volumes in multiple sclerosis. Mult Scler.

[78] Wang JH, Pappas D, De Jager PL, Pelletier D, de Bakker PI, Kappos L (2011). Modeling the cumulative genetic risk for multiple sclerosis from genome-wide association data. Genome Med.

[79] Keshet Y, Seger R (2010). The MAP kinase signaling cascades: a system of hundreds of components regulates a diverse array of physiological functions. Methods Mol Biol.

[80] Chang L, Karin M (2001). Mammalian MAP kinase signalling cascades. Nature.

[81] Shchetynsky K, Protsyuk D, Ronninger M, Diaz-Gallo L-M, Klareskog L, Padyukov L (2015). Gene-gene interaction and RNA splicing profiles of MAP2K4 gene in rheumatoid arthritis. Clin Immunol.

[82] Tuller T, Atar S, Ruppin E, Gurevich M, Achiron A (2013). Common and specific signatures of gene expression and protein-protein interactions in autoimmune diseases. Genes Immun.

[83] GJA t B, Bolk J, `t Hart BA, Laman JD. Multiple sclerosis is linked to MAPKERK overactivity in microglia. J Mol Med. 2021; Available from:. 10.1007/s00109-021-02080-4. 10.1007/s00109-021-02080-4 PMC8313465 33948692

[84] Mass E, Jacome-Galarza CE, Blank T, Lazarov T, Durham BH, Ozkaya N, Pastore A, Schwabenland M, Chung YR, Rosenblum MK, Prinz M, Abdel-Wahab O, Geissmann F (2017). A somatic mutation in erythro-myeloid progenitors causes neurodegenerative disease. Nature.

[85] Kotelnikova E, Kiani NA, Messinis D, Pertsovskaya I, Pliaka V, Bernardo-Faura M, Rinas M, Vila G, Zubizarreta I, Pulido-Valdeolivas I, Sakellaropoulos T, Faigle W, Silberberg G, Masso M, Stridh P, Behrens J, Olsson T, Martin R, Paul F, Alexopoulos LG, Saez-Rodriguez J, Tegner J, Villoslada P (2019). MAPK pathway and B cells overactivation in multiple sclerosis revealed by phosphoproteomics and genomic analysis. Proc Natl Acad Sci U S A.

[86] Krementsov DN, Thornton TM, Teuscher C, Rincon M (2013). The emerging role of p38 mitogen-activated protein kinase in multiple sclerosis and its models. Mol Cell Biol.

[87] Bernardo-Faura M, Rinas M, Wirbel J, Pertsovskaya I, Pliaka V, Messinis DE, Vila G, Sakellaropoulos T, Faigle W, Stridh P, Behrens JR. Prediction of combination therapies based on topological modeling of the immune signaling network in Multiple Sclerosis. Genome Medicine. 2021;13(1):1-6. 10.1186/s13073-021-00925-8 PMC8284018 34271980

[88] Maatz H, Jens M, Liss M, Schafer S, Heinig M, Kirchner M, Adami E, Rintisch C, Dauksaite V, Radke MH, Selbach M, Barton PJR, Cook SA, Rajewsky N, Gotthardt M, Landthaler M, Hubner N (2014). RNA-binding protein RBM20 represses splicing to orchestrate cardiac pre-mRNA processing. J Clin Invest.

[89] Sheikh F, Lyon RC, Chen J (2015). Functions of myosin light chain-2 (MYL2) in cardiac muscle and disease. Gene.

[90] Matyushenko AM, Levitsky DI (2020). Molecular Mechanisms of Pathologies of Skeletal and Cardiac Muscles Caused by Point Mutations in the Tropomyosin Genes. Biochemistry.

[91] Caleshu C, Sakhuja R, Nussbaum RL, Schiller NB, Ursell PC, Eng C, de Marco T, McGlothlin D, Burchard EG, Rame JE (2011). Furthering the link between the sarcomere and primary cardiomyopathies: restrictive cardiomyopathy associated with multiple mutations in genes previously associated with hypertrophic or dilated cardiomyopathy. Am J Med Genet A.

[92] Brody MJ, Hacker TA, Patel JR, Feng L, Sadoshima J, Tevosian SG (2012). Ablation of the cardiac-specific gene leucine-rich repeat containing 10 (Lrrc10) results in dilated cardiomyopathy. PLoS One.

[93] Gupte TM, Haque F, Gangadharan B, Sunitha MS, Mukherjee S, Anandhan S (2015). Mechanistic Heterogeneity in Contractile Properties of α-Tropomyosin (TPM1) Mutants Associated with Inherited Cardiomyopathies*. J Biol Chem.

[94] Huang W, Liang J, Yuan C-C, Kazmierczak K, Zhou Z, Morales A (2015). Novel familial dilated cardiomyopathy mutation in MYL2 affects the structure and function of myosin regulatory light chain. FEBS J.

[95] Herman DS, Lam L, Taylor MRG, Wang L, Teekakirikul P, Christodoulou D, Conner L, DePalma SR, McDonough B, Sparks E, Teodorescu DL, Cirino AL, Banner NR, Pennell DJ, Graw S, Merlo M, di Lenarda A, Sinagra G, Bos JM, Ackerman MJ, Mitchell RN, Murry CE, Lakdawala NK, Ho CY, Barton PJR, Cook SA, Mestroni L, Seidman JG, Seidman CE (2012). Truncations of titin causing dilated cardiomyopathy. N Engl J Med.

[96] Marston S, Montgiraud C, Munster AB, Copeland O, Choi O, Dos Remedios C (2015). OBSCN Mutations Associated with Dilated Cardiomyopathy and Haploinsufficiency. PLoS One.

[97] Marston S (2017). Obscurin variants and inherited cardiomyopathies. Biophys Rev.

[98] McNally EM, Mestroni L (2017). Dilated Cardiomyopathy: Genetic Determinants and Mechanisms. Circ Res.

[99] Boczek NJ, Ye D, Jin F, Tester DJ, Huseby A, Bos JM (2015). Identification and Functional Characterization of a Novel CACNA1C-Mediated Cardiac Disorder Characterized by Prolonged QT Intervals With Hypertrophic Cardiomyopathy, Congenital Heart Defects, and Sudden Cardiac Death. Circ Arrhythm Electrophysiol.

[100] Mouton J, Ronjat M, Jona I, Villaz M, Feltz A, Maulet Y (2001). Skeletal and cardiac ryanodine receptors bind to the Ca(2+)-sensor region of dihydropyridine receptor alpha(1C) subunit. FEBS Lett.

[101] Kanehisa M, Goto S (2000). KEGG: kyoto encyclopedia of genes and genomes. Nucleic Acids Res.

[102] Ather S, Respress JL, Li N, Wehrens XHT (2013). Alterations in ryanodine receptors and related proteins in heart failure. Biochim Biophys Acta.

[103] Yano M, Yamamoto T, Kobayashi S, Matsuzaki M (2009). Role of ryanodine receptor as a Ca2+ regulatory center in normal and failing hearts. J Cardiol.

[104] Moccia F, Lodola F, Stadiotti I, Pilato CA, Bellin M, Carugo S, Pompilio G, Sommariva E, Maione AS (2019). Calcium as a Key Player in Arrhythmogenic Cardiomyopathy: Adhesion Disorder or Intracellular Alteration?. Int J Mol Sci.

[105] Jaffrey SR, Wilkinson MF (2018). Nonsense-mediated RNA decay in the brain: emerging modulator of neural development and disease. Nat Rev Neurosci.

[106] Schwerk C, Schulze-Osthoff K (2005). Regulation of apoptosis by alternative pre-mRNA splicing. Mol Cell.

[107] List M, Alcaraz N, Dissing-Hansen M, Ditzel HJ, Mollenhauer J, Baumbach J (2016). KeyPathwayMinerWeb: online multi-omics network enrichment. Nucleic Acids Res.

[108] Kinsella RJ, Kähäri A, Haider S, Zamora J, Proctor G, Spudich G (2011). Ensembl BioMarts: a hub for data retrieval across taxonomic space. Database.

[109] Kuleshov MV, Jones MR, Rouillard AD, Fernandez NF, Duan Q, Wang Z, Koplev S, Jenkins SL, Jagodnik KM, Lachmann A, McDermott MG, Monteiro CD, Gundersen GW, Ma'ayan A (2016). Enrichr: a comprehensive gene set enrichment analysis web server 2016 update. Nucleic Acids Res.

[110] Signorelli M, Vinciotti V, Wit EC (2016). NEAT: an efficient network enrichment analysis test. BMC Bioinformatics.

[111] Benjamini Y, Hochberg Y (1995). Controlling the False Discovery Rate: A Practical and Powerful Approach to Multiple Testing. J Royal Stat Soc: Ser B (Methodological).

[112] Irimia M, Weatheritt RJ, Ellis JD, Parikshak NN, Gonatopoulos-Pournatzis T, Babor M, Quesnel-Vallières M, Tapial J, Raj B, O’Hanlon D, Barrios-Rodiles M, Sternberg MJE, Cordes SP, Roth FP, Wrana JL, Geschwind DH, Blencowe BJ (2014). A highly conserved program of neuronal microexons is misregulated in autistic brains. Cell.

[113] Dobin A, Davis CA, Schlesinger F, Drenkow J, Zaleski C, Jha S (2013). STAR: ultrafast universal RNA-seq aligner. Bioinformatics.

[114] Van Der Walt S, Chris Colbert S, Varoquaux G. The NumPy array: a structure for efficient numerical computation. arXiv [cs.MS]. 2011; Available from: http://arxiv.org/abs/1102.1523.

[115] McKinney W, Others. Data structures for statistical computing in python. Proceedings of the 9th Python in Science Conference. 2010;445:51–6. 10.25080/Majora-92bf1922-00a.

[116] Hagberg A, Swart P, S Chult D. Exploring network structure, dynamics, and function using networkx. Proceedings of the 7th Python in Science Conference (SciPy2008). 2008;11-15.

[117] Virtanen P, Gommers R, Oliphant TE, Haberland M, Reddy T, Cournapeau D, Burovski E, Peterson P, Weckesser W, Bright J, van der Walt SJ, Brett M, Wilson J, Millman KJ, Mayorov N, Nelson ARJ, Jones E, Kern R, Larson E, Carey CJ, Polat İ, Feng Y, Moore EW, VanderPlas J, Laxalde D, Perktold J, Cimrman R, Henriksen I, Quintero EA, Harris CR, Archibald AM, Ribeiro AH, Pedregosa F, van Mulbregt P, Vijaykumar A, Bardelli AP, Rothberg A, Hilboll A, Kloeckner A, Scopatz A, Lee A, Rokem A, Woods CN, Fulton C, Masson C, Häggström C, Fitzgerald C, Nicholson DA, Hagen DR, Pasechnik DV, Olivetti E, Martin E, Wieser E, Silva F, Lenders F, Wilhelm F, Young G, Price GA, Ingold GL, Allen GE, Lee GR, Audren H, Probst I, Dietrich JP, Silterra J, Webber JT, Slavič J, Nothman J, Buchner J, Kulick J, Schönberger JL, de Miranda Cardoso JV, Reimer J, Harrington J, Rodríguez JLC, Nunez-Iglesias J, Kuczynski J, Tritz K, Thoma M, Newville M, Kümmerer M, Bolingbroke M, Tartre M, Pak M, Smith NJ, Nowaczyk N, Shebanov N, Pavlyk O, Brodtkorb PA, Lee P, McGibbon RT, Feldbauer R, Lewis S, Tygier S, Sievert S, Vigna S, Peterson S, More S, Pudlik T, Oshima T, Pingel TJ, Robitaille TP, Spura T, Jones TR, Cera T, Leslie T, Zito T, Krauss T, Upadhyay U, Halchenko YO, Vázquez-Baeza Y, SciPy 1.0 Contributors (2020). SciPy 1.0: fundamental algorithms for scientific computing in Python. Nat Methods.

[118] Seabold S, Perktold J. Statsmodels: Econometric and statistical modeling with python. Proceedings of the 9th Python in Science Conference. 10.25080/Majora-92bf1922-011.

[119] Fruchterman TMJ, Reingold EM (1991). Graph drawing by force-directed placement. Softw Pract Exp.

[120] Shen S, Park JW, Lu Z-X, Lin L, Henry MD, Wu YN (2014). rMATS: robust and flexible detection of differential alternative splicing from replicate RNA-Seq data. Proc Natl Acad Sci U S A.

[121] Sterne-Weiler T, Weatheritt RJ, Best A, Ha KCH. Whippet: an efficient method for the detection and quantification of 944 alternative splicing reveals extensive transcriptomic complexity. bioRxiv. 2017. 10.1101/158519

[122] Louadi Z. NEASE: A network-based approach for the enrichment of alternative splicing events. Github. 2021. Available from: https://github.com/louadi/NEASE. Accessed 22 Nov 2021.

[123] Louadi Z. NEASE: v.1.1.6. Zenodo; 2021. Available from:. 10.5281/zenodo.5653490.

[124] Louadi Z. NEASE-tutorials: v1.2. Zenodo; 2021. Available from: 10.5281/ZENODO.5562626

