# Supplementary material for: Functional enrichment of alternative splicing events with NEASE reveals insights into tissue identity and diseases
Source: Genome Biol. 2021 Dec 2;22:327. doi: 10.1186/s13059-021-02538-1 (PMC8638120; doi:10.1186/s13059-021-02538-1)
Supplement: Supplementary file 1 — Additional file 1: Tables S1-S3. Table S1. Enrichment of the pathway “Muscle contraction” from Reactome for the exons upregulated in the muscles generated by the NEASE package. Table S2. Enrichment of the pathway “Synaptic vesicle cycle” from KEGG for the exons upregulated in the neural tissues generated by the NEASE package. Table S3. RNA-Seq samples used in the study. Figs. S1-S6. Fig. S1. Node degree distribution of the classic PPI and structurally annotated PPI, the latter contains only interactions with evidence from DDIs and DMIs or residue-level evidence from the co-resolved structure. Fig S2. Network Enrichment Analysis using EviNet webtool for exons upregulated in muscles and neural tissues. Fig. S3. NEASE visualization highlights the interactions of differentially spliced genes with the DCM pathway. Fig. S4. The PSI values of two exon skipping events in the genes TPM1 and DST from the GTEx dataset confirm that both the exons are upregulated in muscles and heart tissues. Fig. S5. The PSI values of two exon skipping events in the genes CLTA and CLTB from the GTEx dataset confirm that both the exons are upregulated in neural tissues. Fig. S6. Pseudocode of NEASE algorithm. [file 13059_2021_2538_MOESM1_ESM.pdf]

Additional file 1: Supplemental figures and tables.

Supplementary Table S1: Enrichment of the pathway "Muscle contraction" from Reactome for the exons up-regulated in the muscles generated by the NEASE package.

Spliced Genes	Affected domain	Gene is known to be in the pathway	Percentage of affected edges associated with the pathway	P-value	Affected binding (edges)
TPM1	PF00261	Yes	10/18	1.35E-15	TPM2<br>MYH8<br>TNNT2<br>MYH6<br>ACTA2<br>TPM4<br>TPM1<br>TNNT1<br>TPM3<br>TNNI1
DST	PF02187	No	1/1	1.13E-02	CALM1
LIMS1	PF00412	No	1/8	8.7E-02	PXN

Supplementary Table S2: Enrichment of the pathway "Synaptic vesicle cycle" from KEGG for the exons up-regulated in the neural tissues generated by the NEASE package.

Spliced Genes	Affected domain	Gene is known to be in the pathway	Percentage of affected edges associated with the pathway	P-value	Affected binding (edges)
ATP6V0A1	PF01496	Yes	7/7	5.85e-17	ATP6V1B1<br>ATP6V0A2<br>ATP6V0D2<br>ATP6V1E1<br>ATP6V0E1<br>ATP6V0D1<br>ATP6V1
CLTA	PF01086	Yes	2/3	6.94e-05	CLTC<br>CLTCL1
CLTB	PF01086	Yes	2/6	3.43e-04	CLTC<br>CLTCL1

Supplementary Table S3. RNA-Seq samples used in the study

Reticulated and mature platelets [1]		
Description	Number of samples	Sequencing depth
Mature platelets	4	15 million single reads
Reticulated platelets	4	20 million single reads
Multiple sclerosis [2]		
Normal-appearing white matter (NAWM)	15	13 - 84 million paired reads
Acute lesion (AL)	20	29 - 301 million paired reads
Dilated Cardiomyopathy (the used data is from [3])		
Heart samples of patients (DCM)	97	90 - 246 million paired reads
Heart samples of healthy donors	108	106 - 231 million paired reads

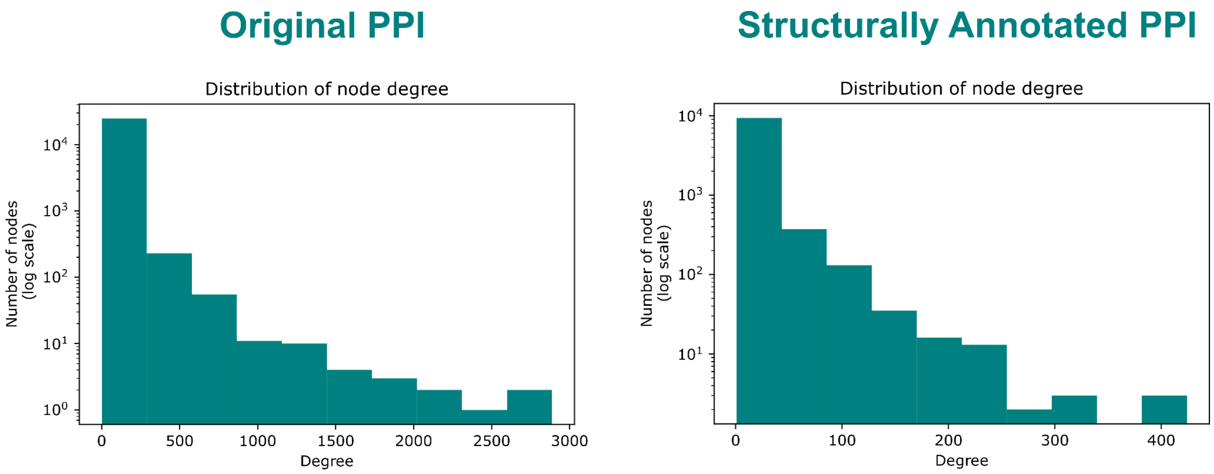

Supplementary Figure S1: Node degree distribution of the classic PPI and structurally annotated PPI, the latter contains only interactions with evidence from DDIs and DMIs or residue-level evidence from the co-resolved structure.

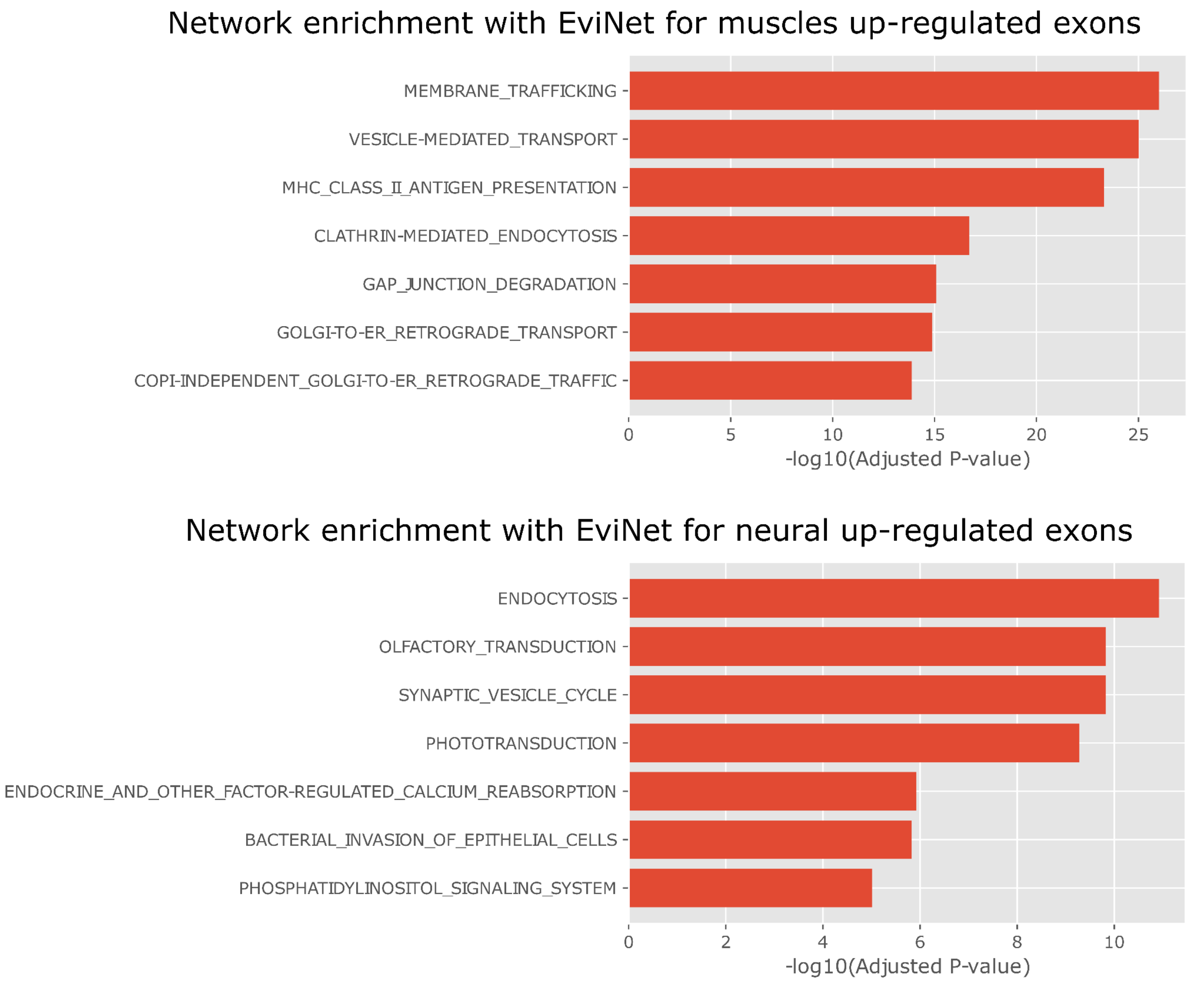

Supplementary Figure S2: Network Enrichment Analysis using EviNet webtool [4] for exons up-regulated in muscles and neural tissues.

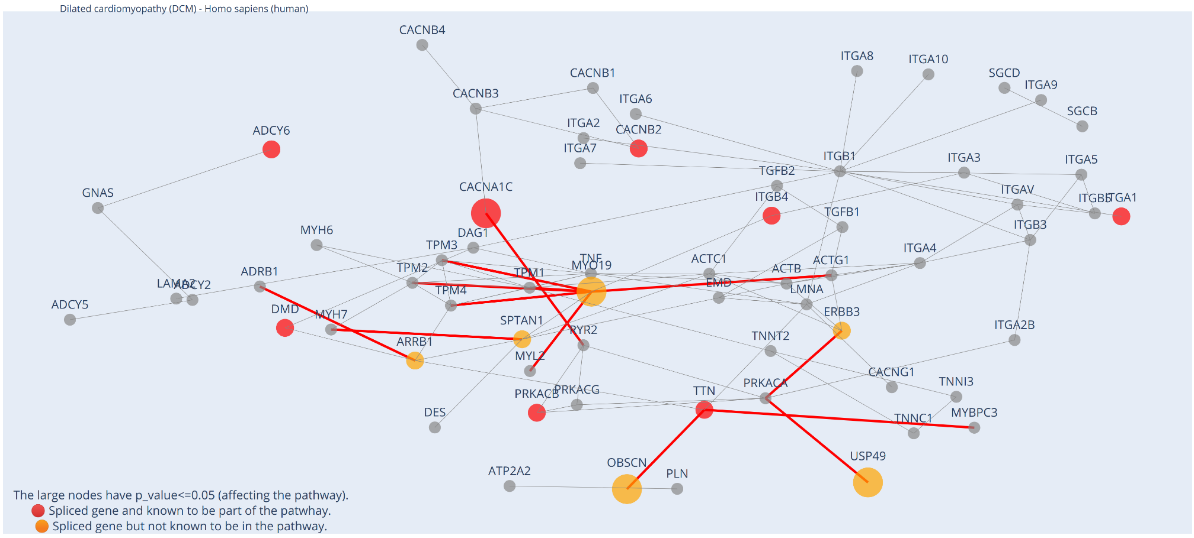

Supplementary Figure S3: NEASE visualization highlights the interactions of differentially spliced genes with the DCM pathway. The gray nodes represent proteins and the red nodes represent differentially spliced genes (both from the pathway). Red edges represent the affected interactions. Orange nodes represent differentially spliced genes that are not in the DCM pathway but have affected interactions with the other genes from it.

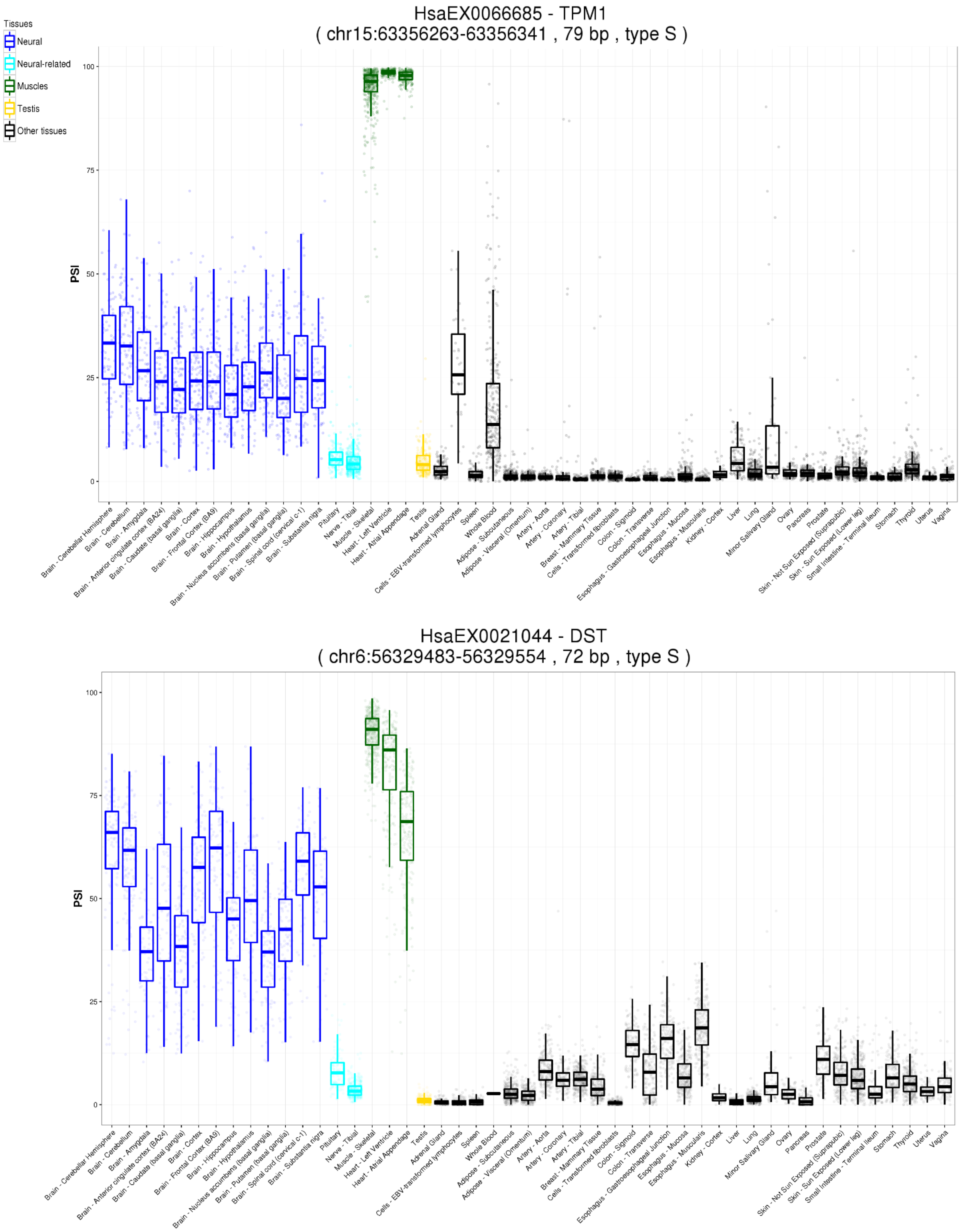

Supplementary Figure S4: The PSI values of two exon skipping events in the genes TPM1 and DST from the GTEx dataset confirm that both the exons are up-regulated in muscles and heart tissues (in green). The original figures are generated from the VastDB website (https://vastdb.crg.eu/, [5]).

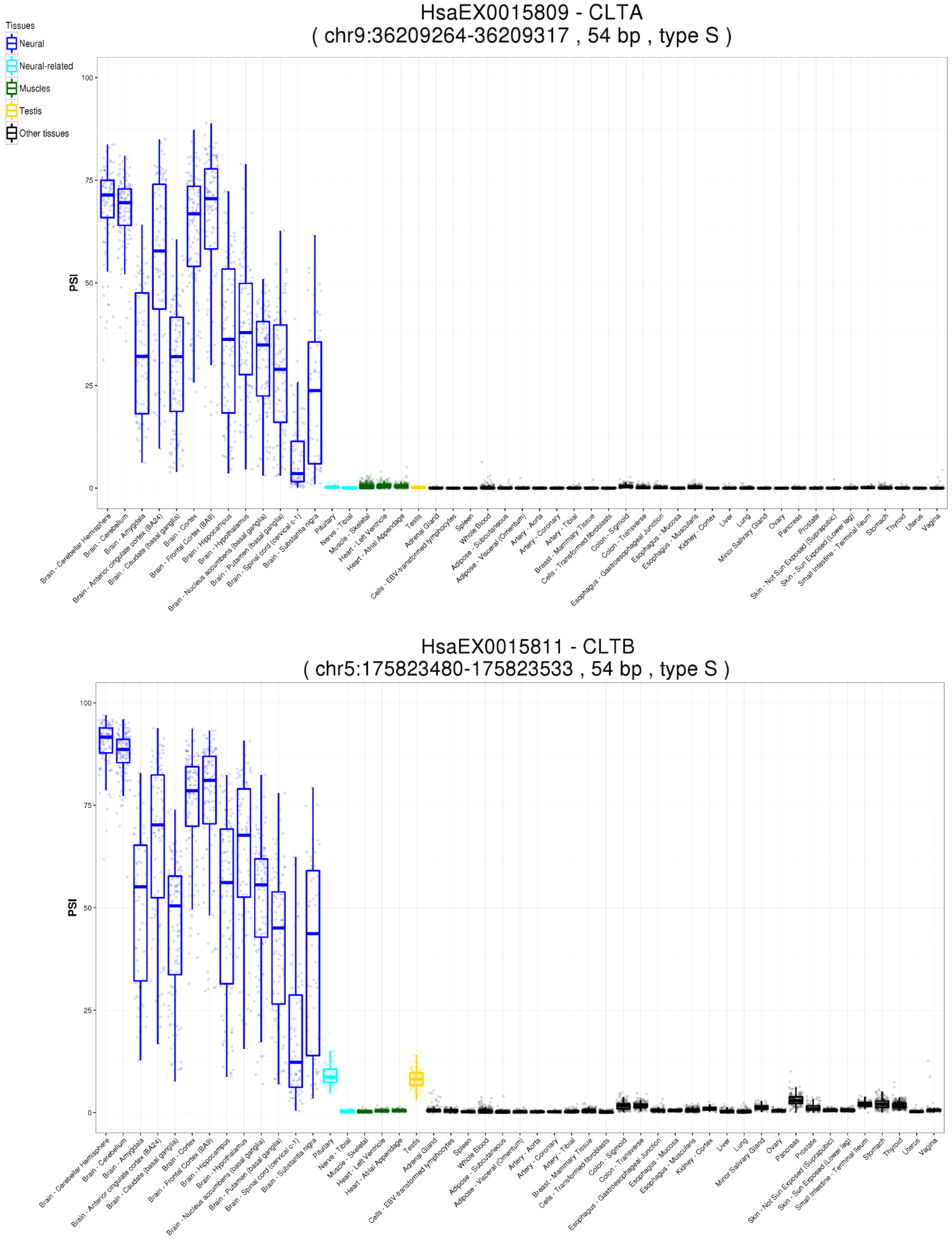

Supplementary Figure S5: The PSI values of two exon skipping events in the genes CLTA and CLTB from the GTEx dataset confirm that both the exons are up-regulated in neural tissues (in blue). The original figures are generated from the VastDB website (https://vastdb.crg.eu/, [5]).

Algorithm 1 Pathway enrichment test.

Construct the structural annotated graph $\mathbf{G}'$.
Calculate $\mathbf{N}$ the total degree of $\mathbf{G}'$.
for Every exon in the submitted query list **do**
  Identify the list of affected domains, motifs or residues.
  Update $\mathbf{e}_{G'}$ affected edges.
end for
Calculate $\mathbf{n}$ the total number of affected edges $\mathbf{e}_{G'}$ from the query.
for Every pathway $\mathbf{P}$ **do**
  Calculate $\mathbf{K}_P$ the pathway degree in the graph $\mathbf{G}'$.
  Calculate $\mathbf{k}$ number affected edges from $\mathbf{e}_{G'}$ that are part of $\mathbf{P}$.
  Calculate the one-sided $\mathbf{pvalue}_P = Hypergeometric(\mathbf{k}, \mathbf{n}, \mathbf{K}_P, \mathbf{N})$
  Calculate the gene-specific p values using **Algorithm 2**.
  Calculate $\mathbf{SCORE}_P$ the adjusted NEASE score using (Eq.: 1).
end for
Correct for multiple testing using Benjamini-Hochberg.
Rank pathways based on adjusted p values or NEASE scores.

Algorithm 2 Gene-specific enrichment test.

For a pathway of interest $\mathbf{P}$ with a degree $\mathbf{K}_P$, this function returns a p value for every gene.
for Every spliced gene $\mathbf{i}$ **do**
  Calculate $\mathbf{n}'$ the number of affected edges from the gene $\mathbf{i}$.
  Calculate $\mathbf{k}'$ the number of edges that are part of $\mathbf{P}$.
  Calculate the gene-specific one-sided $\mathbf{pvalue}_{P_i} = Hypergeometric(\mathbf{k}', \mathbf{n}', \mathbf{K}_P, \mathbf{N})$
end for
Rank genes based on p values.

Supplementary Figure S6: Pseudocode of NEASE algorithm. Algorithm 1: Pathway enrichment test.

Algorithm 2: For every pathway, gene-specific p values are calculated.

References

1. Bongiovanni D, Santamaria G, Klug M, Santovito D, Felicetta A, Hristov M, et al. Transcriptome Analysis of Reticulated Platelets Reveals a Prothrombotic Profile. Thromb Haemost. 2019;119:1795–806.

2. Elkjaer ML, Frisch T, Reynolds R, Kacprowski T, Burton M, Kruse TA, et al. Molecular signature of different lesion types in the brain white matter of patients with progressive multiple sclerosis. Acta Neuropathol Commun. 2019;7:205.

3. Heinig M, Adriaens ME, Schafer S, van Deutekom HWM, Lodder EM, Ware JS, et al. Natural genetic variation of the cardiac transcriptome in non-diseased donors and patients

with dilated cardiomyopathy. Genome Biol. 2017;18:170.

4. Alexeyenko A, Lee W, Pernemalm M, Guegan J, Dessen P, Lazar V, et al. Network enrichment analysis: extension of gene-set enrichment analysis to gene networks. BMC Bioinformatics. 2012;13:226.

5. Tapial J, Ha KCH, Sterne-Weiler T, Gohr A, Braunschweig U, Hermoso-Pulido A, et al. An atlas of alternative splicing profiles and functional associations reveals new regulatory programs and genes that simultaneously express multiple major isoforms. Genome Res. 2017;27:1759–68.
