# Supplementary material for: Functional enrichment of alternative splicing events with NEASE reveals insights into tissue identity and diseases
Source: Genome Biol. 2021 Dec 2;22:327. doi: 10.1186/s13059-021-02538-1 (PMC8638120; doi:10.1186/s13059-021-02538-1)
Supplement: Supplementary file 8 — Additional file 8. Review history. [file 13059_2021_2538_MOESM8_ESM.docx]

Review History

**First round of review**

**Reviewer 1**

**Were you able to assess all statistics in the manuscript, including the appropriateness of statistical tests used?**

Yes: Unclear if multiple testing correction was applied. More detail description statistical tests used in the manuscript is required.

**Were you able to directly test the methods?**

Yes

**Comments to author:**

Summary This manuscript details NEASE (Network-based Enrichment Method for Alternative splicing Events), a tool that characterises alternative splicing (AS) events using protein-protein (PPI) and domain-domain interactions (DDI). This is a novel python package developed to identify enriched pathways from functionally significant domain changes due to alternative splicing. First, NEASE identifies domains effected by AS by using dPSI values supplied as input. Then PPI and DDI are combined into an integrated network, detailed in previous papers, to identify protein interaction partners of the AS events. Finally, NEASE applies the hypergeometric test for enrichment of terms. This prioritises genes affected by the interactome rather than the industry standard of gene-level enrichment. The NEASE score quantifies the total number of connected genes from spliced genes and the log p-value. The manuscript then applies NEASE in four tutorials to identify differentially spliced genes between healthy and disease cohorts. This manuscript introduces an interesting methodological approach for interrupting results from RNA-seq alternative splicing analysis and test cases. However, the manuscript only briefly describes the developed method. This makes the algorithm difficult to assess, and compare to previous approaches.

Major changes.

1) The authors must provide a more thorough description of the algorithm they have developed. This includes both a more detailed description in the text and more clear figures. Figure 1 is unclear and needs to be revamped to provide clearer overview of the method.

2) Event selection - it is unclear from manuscript whether the algorithm includes all exons in analysis or filters out certain exons, for example, based on whether exon is divisible by 3. If no filter is implemented then this is likely to lead to high false positives. Authors should include and/or describe filters that remove exons unlikely to form part of functional proteins. This includes exons that may disrupt protein folding. It is also unclear how authors deal with multiple alternative splicing events within a gene.

3) Does the algorithm only analyse cassette exons or also alternative 5' or 3' and intron retention events?

4) Benchmarking - the authors suggest that using their domain approach provides unique and meaningful biological insights. However, they provide no evidence that it is, for example superior to just using GO-term analysis of differential exons. Or previously developed tools for the evaluation of alternative splicing differential analysis. The authors need to benchmark their approach against previous approaches used in the field. Suggestions include performance comparison to PPI only, GO and protein coding exons. Also, bootstrapping could be applied.

5) Interactions used. DDI are the dominant PPI in databases. However, extensive evidence suggests that alternative exons preferentially encode intrinsically disordered regions (e.g. Romero et al.). The reviewers should include data from resources annotating these interactions (such as ELM and Domino) to ensure these interactions are included

6) Statistics. Do the authors correct for multiple testing?

7) Protein-protein interactions chosen - EBI Intact also includes annotated information of residues involved in PPIs. This resource should also be included.

8) Tutorials on GitHub - The GitHub tutorials (https://github.com/louadi/NEASE) and pip installation are easy to use and understand. However, the authors should supply a more detailed description of the input datasets and functions of the NEASE package. Specifically, the composition standard input data frame needs to be provided. The authors should describe all the functions and a short description of the flags. This should be included in the README.md. Description of standard input should also be included in main text

9) The authors need to ensure the input is viable with majority of splicing algorithms (e.g. at least rMATs, VAST-TOOLS, MAJIQ, MISO and Whippet).

10) In Figure 2B and 4A, the high percentage of domains with unknown interactions is not mentioned or explained in the text. From 31% to 41% of domains with unknown interactions is a large proportion of domains excluded from the functional analysis.

10) The author must provide evidence their approach does not bias towards highly studied "hub" genes, as this is likely to be the majority of annotated domain-domain interactions.

Minor comments

1) For the tutorials, during the VastDB pre-processing analysis the hg19.tab file was utilised. However, hg38 in RNA-seq analysis. Please be consistent

2) Line 439 - authors must provide more details about test and how they modified it.

3) In the text the Figure 2A is described as "heatmap shows two distinct clusters, where neural-specific and muscle-specific (merged with heart-specific) exons are dominant." However, these clusters are not clear upon visual inspection

**Reviewer 2**

**Were you able to assess all statistics in the manuscript, including the appropriateness of statistical tests used?**

No

**Were you able to directly test the methods?**

Yes

**Comments to author:**

In their manuscript 'Functional enrichment of alternative splicing events with NEASE reveals insights into tissue identity and diseases' Tsoy and colleagues describe a bioinformatics tool to investigate the potential functionality of a group of co-regulated alternative exons identified by RNA-Seq. Such a tool would certainly be a very valuable addition for the analysis of alternative splicing, as the standard method as of today, is to use GO term analysis of the alternatively spliced genes. This method is not focused in the alternative exon but rather on the whole gene and the results may therefore be misleading. The tool presented here uses the protein sequence encoded by alternative exons, finds known protein domains that are altered by alternative splicing and then predicts functionality based on protein-protein or domain-domain interactions. This tool would close an important gap to analyze the functional impact of global alternative splicing changes in different condition, e.g. comparing different tissues. However, the work is entirely based on bioinformatics and the predictions have to be validated. This has to start with the validation of the groups of alternative exons that are defined as being muscle specific (or neuron specific etc.).

What is the validation rate of randomly chosen (not only the top targets) exons that are classified as tissue specific and that are then used for the prediction pipeline? At least 10 exons should be tested (given the very low number of exons that goes in the pipeline, I would actually validate all of them). Even a validation rate of 50% would be problematic, as it would mean that 50% of the exons used for the prediction are false positives. And then at least one of the predicted changes in PPI or pathway activity should be validated experimentally.

Another problem is the very low number of exons that finally goes in the prediction (and also please provide PSI values for the different tissues). While I appreciate that this is focused on exons that affect protein domains, this is hardly a global analysis and would strongly underestimate the functional impact of alternative splicing. And this low number will make predictions unreliable, as some, probably even a single additional event(s), may substantially alter the enrichment scores. Altering other parts of proteins than known domains can have a substantial impact on the proteins function and is neglected here.

In addition, there are hundreds of NMD inducing exons. They will regulate the whole transcript, which then justifies the use of classical GO term analysis. To increase the impact of this pipeline, additional AS events should be integrated to increase the number of events that go into the pipeline and that predictions are build on.

**Authors Response**

**Point-by-point responses to the reviewers’ comments:**

Reviewer #1:

Summary

This manuscript details NEASE (Network-based Enrichment Method for Alternative splicing Events), a tool that characterises alternative splicing (AS) events using protein-protein (PPI) and domain-domain interactions (DDI). This is a novel python package developed to identify enriched pathways from functionally significant domain changes due to alternative splicing.

First, NEASE identifies domains effected by AS by using dPSI values supplied as input. Then PPI and DDI are combined into an integrated network, detailed in previous papers, to identify protein interaction partners of the AS events. Finally, NEASE applies the hypergeometric test for enrichment of terms. This prioritises genes affected by the interactome rather than the industry standard of gene-level enrichment. The NEASE score quantifies the total number of connected genes from spliced genes and the log p-value. The manuscript then applies NEASE in four tutorials to identify differentially spliced genes between healthy and disease cohorts.

This manuscript introduces an interesting methodological approach for interrupting results from RNA-seq alternative splicing analysis and test cases. However, the manuscript only briefly describes the developed method. This makes the algorithm difficult to assess, and compare to previous approaches.

*We thank the reviewer for evaluating our method and for suggesting multiple data sources to extend the method coverage.*

Major changes.

1) The authors must provide a more thorough description of the algorithm they have developed. This includes both a more detailed description in the text and more clear figures. Figure 1 is unclear and needs to be revamped to provide clearer overview of the method.

*Following the reviewer’s request, we extended the description of the algorithm in the Method section by explaining Figure 1 examples step-by-step. We also added detailed pseudocode of the algorithm as aN Additional file 1: Supplementary Figure S6.*

2) Event selection - it is unclear from manuscript whether the algorithm includes all exons in analysis or filters out certain exons, for example, based on whether exon is divisible by 3. If no filter is implemented then this is likely to lead to high false positives. Authors should include and/or describe filters that remove exons unlikely to form part of functional proteins. This includes exons that may disrupt protein folding. It is also unclear how authors deal with multiple alternative splicing events within a gene.

*The only strictly necessary filtering for running NEASE (in differential splicing analysis case) is the dpsi value and its corresponding p-value. Additional filtering steps should be guided by the user and the specific biological question at hand. In our example, we decided to filter for tissue-specific exons but we feel that such a choice should be left to the user and that generally applicable filter rules are not easily defined.*

*For instance, while it is true that many AS events do not necessarily result in functional proteins, these can still be functionally relevant events and lead to e.g. downregulation of the whole gene expression (McGlincy and Smith 2008 10.1016/j.tibs.2008.06.001). Our logic here is that a spliced domain should be considered functionally relevant irrespective of whether it leads to an alternative isoform or if nonsense-mediated decay leads to an unfoldable protein. In the latter case, we are less certain about the rest of the protein but confident that the domain in consideration is affected, and so are its interactions. In this way, we improve the signal-to-noise ratio by only including the interactions that are most likely affected. We now provide further explanations for these design decisions in the Discussion section. Nevertheless, the exons that are not divisible by 3 have indeed a higher chance to cause NMD. In that case, classical gene set enrichment is justified. For this reason, we added the support of classic gene set enrichment for such events (or optionally to all events). It is also up to the user to filter these events in case he/she wants to focus on events with a high potential of coding.*

*For the second part of the question: in the case of multiple AS events affecting the same genes, we consider every event individually and identify all protein features. We also added these details to the manuscript “the python package sections”.*

3) Does the algorithm only analyse cassette exons or also alternative 5' or 3' and intron retention events?

*In our analysis and the example datasets, we focused on exon skipping events since they are the most studied types and their impact on protein features is more straightforward to predict. However, NEASE’s algorithm is in general agnostic to the event type. The user can control which events are considered in preprocessing of NEASE’s input by adding the coordinate of the spliced region(s). For instance, the coordinate of an alternative 3’ splice-site. We added these details to the manuscript.*

4) Benchmarking - the authors suggest that using their domain approach provides unique and meaningful biological insights. However, they provide no evidence that it is, for example superior to just using GO-term analysis of differential exons. Or previously developed tools for the evaluation of alternative splicing differential analysis. The authors need to benchmark their approach against previous approaches used in the field. Suggestions include performance comparison to PPI only, GO and protein-coding exons. Also, bootstrapping could be applied.

*We appreciate the concern of the reviewer. In the five use cases we presented, we extensively compared NEASE with classical “gene-level enrichment”. For example, In figures 2 and 4, we show the enrichment obtained by classical enrichment analysis (Fisher’s exact test) using spliced genes and discuss how NEASE offers unique biological insights. It should be noted that a gold standard for functional enrichment analysis currently does not exist as highlighted by Mitrea et al., among others (https://doi.org/10.3389/fphys.2013.00278). Recent attempts towards such a gold standard rely on annotated disease genes as ground truth (see e.g. Geistlinger et al. https://doi.org/10.1093/bib/bbz158). These disease gene annotations are naturally incomplete and, importantly, may not include many of the genes affected by alternative splicing which is a process that is understudied compared to disease-associated gene expression changes. Extending our knowledge of disease-associated alternative splicing events motivated the development of NEASE in the first place and, in contrast to classical gene-level enrichment, NEASE can identify new candidates or biomarkers that are not yet annotated and can lead to new biological insights. NEASE achieves this by focusing on the interactions of the gene (edges) rather than the genes themselves (nodes). We show that this approach reveals disease-associated genes in dilated cardiomyopathy which were not initially part of the KEGG disease pathway but confirmed in recent literature. Such genes wouldn’t be considered in the classic overrepresentation test (one-sided Fisher test).*

*A comparison with alternative methods is also prohibited by the unique approach taken by NEASE. We are not aware of any other functional enrichment method that operates at the exon rather than the gene level. Existing tools allow investigating AS-driven changes in an explorative fashion but tools for systematic analysis of functional effects of AS are lacking.*

*In contrast to a comparison against classical enrichment methods, the suggested comparison to a PPI-only-based approach is interesting, since it yields insights into the usefulness of exon-specific PPIs used in NEASE, which we obtain by integrating domain-domain interactions, eukaryotic linear motifs, and structural data from PDB. To test this, we performed network enrichment analysis (EviNet: doi.org/10.1093/nar/gky485, https://www.evinet.org) on the heart/muscle-specific and neural-specific exons. Similar to our approach, EviNet considers enrichment of edges between a set of active nodes (spliced genes in our case) and a pathway using a permutation test. However, in contrast to our method, this method looks at all edges of the active genes, rather than focusing on those interactions that are likely affected by exon skipping (spliced domains and motifs). For a fair comparison, we run EviNet on the same PPI database (BioGrid) and on the same pathways databases (Reactome and KEGG). As shown Additional file 1: Supplementary Figure S2, the pathways obtained from the PPI enrichment were very similar to the ones found in classical gene set enrichment, which suggests that our exon-specific specific approach helps narrow down the exact complexes/pathways affected by AS and reduce false positives. This additional comparison was added to the manuscript.*

*We also thank the reviewer for the valuable suggestion to consider bootstrapping. To further validate the method from other possible biases such as hubs and literature biases, we run a permutation test by randomly sampling exon skipping events present in neural and muscle tissues and compare the obtained enrichment (p values) with the one obtained from neural-specific and muscle-specific exons, respectively. For a more realistic scenario, our background set of exons are not completely random but we rather select exons skipping events that can actually be found in these tissues. The empirical p-values are obtained from a distribution of p-values, by asking how likely is it to obtain a p-value as low or more extreme than the one reported by NEASE in the original set of tissue-specific exons. The empirical p-values are 0.0008 and 0.0001 for neural exons and muscles up-regulated, respectively. These results further demonstrate the robustness and significance of our analysis.*

5) Interactions used. DDI are the dominant PPI in databases. However, extensive evidence suggests that alternative exons preferentially encode intrinsically disordered regions (e.g. Romero et al.). The reviewers should include data from resources annotating these interactions (such as ELM and Domino) to ensure these interactions are included

*We thank the reviewer for the valuable suggestion and extended NEASE coverage to include Linear Motif mediating interactions from the ELM databases. We mapped 2,175 instances of ELMs to their corresponding coding exons and further identified interactions mediated by these linear motifs. Next, we identified PPI interactions that are possibly mediated by them using domain-motif interactions (DMI) from the same resource. In total, we found 3,926 PPIs with at least one evidence from DMI. We have re-done all the analysis on the 4 datasets again and discussed the new affected motifs by AS. The results largely agree with the DDIs analysis that was done initially as shown in the updated results sections.*

*For instance, in exons up-regulated in the neural tissues, we found a new exon skipping events overlapping with the motif “Adaptin binding Endosome- Lysosome- Basolateral sorting signals” in the gene ATP2B1. Which is another gene that is also linked to the “Synaptic vesicle cycle” pathway.*

*The NEASE python package now provides 2 additional functions intended for ELMs. The first one shows all affected ELMs from the list of exons with their IDs and external link for information. The second function provides possible interactions affected by each of these linear motifs with a list of interaction partners.*

6) Statistics. Do the authors correct for multiple testing?

*NEASE correct for multiple testing at the pathway enrichment stage using Benjamini-Hochberg. We also initially reported the adjusted p values in all tables. Details are available in the Method section.*

7) Protein-protein interactions chosen - EBI Intact also includes annotated information of residues involved in PPIs. This resource should also be included.

*In a similar way to ELMs, we extended the coverage of the structurally annotated interactions with residue-level interactions that were mapped to their exons. The data used here was initially obtained from the PDB database (Berman et al. 2000:https://doi.org/10.1093/nar/28.1.235) and used in our previous publication (Louadi et al. 2020: https://doi.org/10.1093/nar/gkaa768) where we explain in detail the pre-processing steps. In summary 49,835 coding exons were found to encode for residues involved in the PPIs. That new release of the NEASE tool now integrates residue-level interactions in addition to motifs and domains interactions. We have also re-done the statistics using the new data and graph. We found new events/genes candidates that were not initially found from the DDI study and that also confirm our previous results.*

*One interesting example, in multiple Sclerosis we confirmed the previous interactions of the gene ATP2B1 and found new potential affected interaction with the gene NPTN that is also a part of “Neurotransmitter receptors” and “Transmission across Chemical Synapses” which further confirmed our initial results.*

8) Tutorials on GitHub - The GitHub tutorials (https://github.com/louadi/NEASE) and pip installation are easy to use and understand. However, the authors should supply a more detailed description of the input datasets and functions of the NEASE package. Specifically, the composition standard input data frame needs to be provided. The authors should describe all the functions and a short description of the flags. This should be included in the README.md. Description of standard input should also be included in main text

*We thank and appreciate the reviewer for testing and evaluating our package. We now extended the description on the main GitHub page (https://github.com/louadi/NEASE) and included a list of all available functions. The description of the standard input was already described in the manuscript “Python package section”. We also extended the tutorials by adding new functions to the package that are intended to explore ELM and residues involved in PPIs (https://github.com/louadi/NEASE-tutorials).*

9) The authors need to ensure the input is viable with majority of splicing algorithms (e.g. at least rMATs, VAST-TOOLS, MAJIQ, MISO and Whippet).

*In addition to the standard input, the package now supports the output of the following tools: Whippet, MAJIQ, rMATS and DEXSeq.*

10) In Figure 2B and 4A, the high percentage of domains with unknown interactions is not mentioned or explained in the text. From 31% to 41% of domains with unknown interactions is a large proportion of domains excluded from the functional analysis.

*We thank the reviewer for this remark. In our database, not all domains (and motifs) have known interactions because they either have no DDIs or their corresponding genes have no known PPIs. Our knowledge of high-confidence DDIs and co-resolved structure is still incomplete, as we and others have previously discussed (Louadi et al. 2020: https://doi.org/10.1093/nar/gkaa768, Will and Helms: https://doi.org/10.1093/bioinformatics/btv620. We believe that with the expected improvement of structural annotations over time, the impact of our method will increase significantly.*

*Another reason is the fact that some domains are not participating in the PPI but rather regulate binding with DNA or RNA. This is also another interesting regulation of AS but out of the scope of our current approach. Nevertheless, our python package reports all affected domains and motifs to the user with a link to get more information about them and to visualize transcript variants with or without the domain. However, the enrichment and statistical analysis are limited to high-confidence DDIs, DMI, and PDB structures. We extended the discussion section with these details.*

10) The author must provide evidence their approach does not bias towards highly studied "hub" genes, as this is likely to be the majority of annotated domain-domain interactions.

*In order to check if the structurally annotated PPI is more biased to hubs than the standard PPI network, we compute the histogram of the two graphs. As shown in the figures (Additional file 1: Supplementary Figure S1), the distribution of the PPI after filtering (DDI, ELM, PDB edges only) is similar to the original PPI with an overall smaller degree. The maximum degree in the structural graph is equal to 424, compared to 2886 in the full PPI. This observation shows that adding DDIs decreases the overall number of edges but doesn’t increase the bias toward hub genes.*

*The figures were added to the supplementary (Additional file 1: Supplementary Figure S1) and mentioned in the text.*

*In addition, NEASE performs a number of steps to avoid biases to hubs in the analysis. Firstly, for individual genes, we perform a Fisher exact test on node level to test if a gene has a significant number of interactions related to the pathway from all affected ones (Algorithm 2 in the pseudocode). In this way, hub nodes will have a high p-value compared to the rest of the nodes if they have only a few edges in the pathway. At the pathway level, the NEASE score aims to scale pathways with fewer hubs (not significant nodes). Using these measurements, the user can focus on the most significant and less biased results.*

Minor comments

1) For the tutorials, during the VastDB pre-processing analysis the hg19.tab file was utilised. However, hg38 in RNA-seq analysis. Please be consistent

*We appreciate the concern and agree that hg38 should be used consistently where possible. However, we performed the VastDB analysis using hg19 to match the GTEx data which was mapped to the same reference. This was necessary to replicate the results directly in two independent datasets without introducing potential biases caused by coordinate liftover. Note that this validation step was also requested by reviewer 2.*

2) Line 439 - authors must provide more details about test and how they modified it.

*We now extended the description of the test and explained the difference with the previous one. The main difference is that the original test checks if a set of active nodes is significantly connected to a pathway module. In our test, we consider only the interactions likely affected by AS. Furthermore, the background of our test is only structurally annotated PPIs instead of the full PPI.*

3) In the text the Figure 2A is described as "heatmap shows two distinct clusters, where neural-specific and muscle-specific (merged with heart-specific) exons are dominant." However, these clusters are not clear upon visual inspection

*We updated figure 2A and added clusters labels to help to guide the readers.*

Reviewer #2:

In their manuscript 'Functional enrichment of alternative splicing events with NEASE reveals insights into tissue identity and diseases' Tsoy and colleagues describe a bioinformatics tool to investigate the potential functionality of a group of co-regulated alternative exons identified by RNA-Seq. Such a tool would certainly be a very valuable addition for the analysis of alternative splicing, as the standard method as of today, is to use GO term analysis of the alternatively spliced genes. This method is not focused in the alternative exon but rather on the whole gene and the results may therefore be misleading. The tool presented here uses the protein sequence encoded by alternative exons, finds known protein domains that are altered by alternative splicing and then predicts functionality based on protein-protein or domain-domain interactions. This tool would close an important gap to analyze the functional impact of global alternative splicing changes in different condition, e.g. comparing different tissues.

*We thank the reviewer for evaluating our method and for the positive feedback.*

However, the work is entirely based on bioinformatics and the predictions have to be validated. This has to start with the validation of the groups of alternative exons that are defined as being muscle specific (or neuron specific etc.). What is the validation rate of randomly chosen (not only the top targets) exons that are classified as tissue specific and that are then used for the prediction pipeline? At least 10 exons should be tested (given the very low number of exons that goes in the pipeline, I would actually validate all of them). Even a validation rate of 50% would be problematic, as it would mean that 50% of the exons used for the prediction are false positives. And then at least one of the predicted changes in PPI or pathway activity should be validated experimentally.

*While we fully agree that experimental validation of computational findings is paramount, we think that in the case of NEASE validating single or even multiple splicing events will not prove the validity of our method which is intended for the interpretation of experimental results on a systems biology / functional level. In the same way, reported biological or molecular function from GSEA can not be confirmed by checking the expression of an individual gene. In-depth functional studies of the underlying molecular mechanisms are necessary to study these individual aspects of these pathways and we feel that these are beyond the scope of this manuscript. We also note that, like all enrichment methods, we suffer from the current lack of ground truth data for evaluating computational enrichment methods. This issue has been noted in multiple studies (see our response to reviewer 1 in 1.4 - benchmarking).*

*Given this, we think that the best feasible evaluation for such an approach is to show that our interpretation is biologically plausible across multiple data sets. To this end, we applied NEASE in 5 different scenarios including tissue-specific exons, platelet development, and two different diseases (Multiple Sclerosis and Dilated Cardiomyopathy). We further highlight in detail our unique focus on alternative splicing not covered by comparable tools at all.*

*Nevertheless, we can indeed strengthen our results by showing that they are non-random and statistically significant. One approach to validate tissue-specific exons is by looking at other large-scale and independent datasets like GTEx. VastDB includes the quantification of PSI values from 8,378 samples (49 tissues and 543 individuals) from GTEx version 6 on their website (https://vastdb.crg.eu/). We manually investigated the PSI values for the most critical exons in neural and muscle-specific analysis (exons with best p values at enrichment stage). We found that the exons are clearly up-regulated in their respective tissues as shown in Additional file 1 Supplementary Figures S4 and S5 .*

*Another approach to check the validity of the enriched pathways and the tissue-specific exons is by performing permutation tests. Here, our null hypothesis is that the tissue-specific exons are not any different than a random set of exons in terms of relevant enrichment obtained. For a more realistic scenario, our background set of exons are not completely random but we rather select exons skipping events that can actually be found in these tissues. The empirical p-values are obtained from a distribution of p-values, by asking how likely is it to obtain a p-value as low or more extreme than the one reported by NEASE in the original set of tissue-specific exons. The empirical p-values are 0.0008 and 0.0001 for neural exons and muscles up-regulated, respectively. These results further demonstrate the robustness and significance of our analysis.*

Another problem is the very low number of exons that finally goes in the prediction (and also please provide PSI values for the different tissues). While I appreciate that this is focused on exons that affect protein domains, this is hardly a global analysis and would strongly underestimate the functional impact of alternative splicing. And this low number will make predictions unreliable, as some, probably even a single additional event(s), may substantially alter the enrichment scores.

Altering other parts of proteins than known domains can have a substantial impact on the function of the protein and is neglected here. In addition, there are hundreds of NMD inducing exons. They will regulate the whole transcript, which then justifies the use of classical GO term analysis.

To increase the impact of this pipeline, additional AS events should be integrated to increase the number of events that go into the pipeline and that predictions are build on.

*We agree with the reviewer about these limitations and the fact that the coverage of the considered events is low. In order to increase the impact of NEASE, we added more protein features that could mediate interactions such as linear motifs and individual residues from co-resolved structures in the PDB databases. The methodology of including these resources is answered in reviewer 1 response and explained in the manuscript. We have also redone the experiments on the 4 datasets. We also now provide the PSI values for the different tissues as an additional file.*

*One of the interesting examples, in exons up-regulated in the neural tissues, we found a new exon skipping events overlapping with the motif “Adaptin binding Endosome- Lysosome- Basolateral sorting signals” in the gene ATP2B1. Which is another gene also associated to “Synaptic vesicle cycle”. In another example in the multiple sclerosis dataset we used co-resolved structure from PDB database to confirm the previous interactions of the gene ATP2B1 and found a new potentially affected interaction with the gene NPTN that is also a part of the “Neurotransmitter receptors” and “Transmission across Chemical Synapses” pathways. These additional resources confirmed and provided additional insights to our results and will certainly be valuable for the future users of NEASE since there are multiple pieces of evidence that AS affects disorder regions quite often.*

*We also agree (as we discussed before) that the case of NMD is similar to gene expression regulation and classical enrichment would make sense there. NEASE now also provides the classical gene set enrichment for non-symmetrical alternative exons that are likely to cause NMD (Magen and Ast 2005: https://doi.org/10.1093/nar/gki858). In line with this, we previously argued that NEASE is complementary to GSEA and other gene-level approaches and they should be used together for a global view.*

*At the moment, the functional characterization of all exons is far from complete. We think that an increase of annotations at isoform-level and structural information will greatly raise the usefulness of NEASE in the future. Additionally, further progress is needed to link transcriptomics with proteomics for better characterization and understanding of the impact of individual AS events. Nevertheless, NEASE is still the only functional enrichment method that can be used to characterize AS events and exons systematically instead of only considering affected genes. We have also shown that the obtained biological insights can be very valuable and complementary to gene-level analysis. We extended the discussion to include all these limitations*

*In summary, even though NEASE currently doesn’t cover all exons, NEASE offers an unprecedented opportunity to explore in detail potentially protein complexes and pathways rewired by AS.*

**Second round of review**

**Reviewer 1**

We thank the authors for answering our concerns. Our only remaining concern is in reference to including non-in-frame exons within a protein analysis. The authors should automatically remove non-in-frame exons from their analysis as default. An option to include all exons in analysis could be included but having it as default is troublesome.

**Reviewer 2**

The revised version of the manuscript ‘Functional enrichment of alternative splicing events with NEASE reveals insights into tissue identity and diseases’ is markedly improved. I still think that experimental validation of splicing events would further improve the confidence in the presented pipeline, but the authors do present some data to address this point to certain degree. What remains is the concern about the very low number of splicing events that are used; for muscle and neuron-specific splicing these are less than 20 events, which makes it very hard to draw meaningful conclusions and the pipeline should not pretend to perform a global analysis. If publication is considered, this should be discussed very prominently and the exact numbers of exons that conclusions/enrichments are based on should be mentioned in all figure legends of directly in the figures.

**Authors Response**

**Point-by-point responses to the reviewers’ comments:**

We thank the reviewers for their thoughtful remarks. We addressed their concerns and the remaining editorial concerns in the newest version of the manuscript. We also corrected minor typos and mislabelled additional files.

Reviewer #1:

We thank the authors for answering our concerns.

Our only remaining concern is in reference to including non-in-frame exons within a protein analysis. The authors should automatically remove non-in-frame exons from their analysis as default. An option to include all exons in the analysis could be included but having it as default is troublesome.

*We extended the Python package to include two automatic filtering of exons. The first option, run by default and it is based on VastDB predictions of the exons that are likely to cause disruption on the open reading upon inclusion/exclusion of the exons that are known to produce “Non-Coding” transcripts (Tapial, J., 2017). The second is optional and removes all exons that are not divisible by 3, since these exons have higher chance to results in Non-coding transcripts. The user can optionally turn these two parameters off. We explained the option in the manuscript and the GitHub page (https://github.com/louadi/NEASE).*

Reviewer #2: The revised version of the manuscript ‘Functional enrichment of alternative splicing events with NEASE reveals insights into tissue identity and diseases’ is markedly improved. I still think that experimental validation of splicing events would further improve the confidence in the presented pipeline, but the authors do present some data to address this point to certain degree. What remains is the concern about the very low number of splicing events that are used; for muscle and neuron-specific splicing these are less than 20 events, which makes it very hard to draw meaningful conclusions and the pipeline should not pretend to perform a global analysis. If publication is considered, this should be discussed very prominently and the exact numbers of exons that conclusions/enrichments are based on should be mentioned in all figure legends of directly in the figures.

*We appreciate the concern of the reviewer. We previously reported the number and percentage of events considered in every dataset both in the text and figures (e.g: figure 2B and figure 4). We now also include it in the figures legends and we extended the discussion to highlight this limitation.*

*For future users, the Python package provides the exact statistics and generates automatic plots to highlight the number of events that are considered for the enrichment. For instance, figure 2B is directly generated from the package using the function “get_stats”.*
